# Oscillating Fluid Flow Activated Osteocyte Lysate‐Based Hydrogel for Regulating Osteoblast/Osteoclast Homeostasis to Enhance Bone Repair

**DOI:** 10.1002/advs.202204592

**Published:** 2023-04-05

**Authors:** Liyuan Zheng, Disheng Zhou, Feier Ju, Zixuan Liu, Chenzhi Yan, Zhaoxia Dong, Shuna Chen, Lizhi Deng, Szehoi Chan, Junjie Deng, Xingding Zhang

**Affiliations:** ^1^ Shenzhen Key Laboratory for Systems Medicine in Inflammatory Diseases School of Medicine, Shenzhen Campus of Sun Yat‐Sen University Sun Yat‐sen University Shenzhen 518106 P. R. China; ^2^ Joint Centre of Translational Medicine The First Affiliated Hospital of Wenzhou Medical University Wenzhou Zhejiang 325000 P. R. China; ^3^ Joint Centre of Translational Medicine Wenzhou Institute University of Chinese Academy of Sciences Wenzhou Zhejiang 325000 P. R. China; ^4^ Zhejiang Engineering Research Center for Tissue Repair Materials Wenzhou Institute University of Chinese Academy of Sciences Wenzhou Zhejiang 325000 P. R. China

**Keywords:** bone repair, hydrogel, oscillating fluid flow, osteoblasts, osteoclasts, osteocytes

## Abstract

As major regulators on bone formation/resorption in response to mechanical stimuli, osteocytes have shown great promise for restoring bone injury. However, due to the unmanageable and unabiding cell functions in unloading or diseased environments, the efficacy of osteogenic induction by osteocytes has been enormously limited. Herein, a facile method of oscillating fluid flow (OFF) loading for cell culture is reported, which enables osteocytes to initiate only osteogenesis and not the osteolysis process. After OFF loading, multiple and sufficient soluble mediators are produced in osteocytes, and the collected osteocyte lysates invariably induce robust osteoblastic differentiation and proliferation while restraining osteoclast generation and activity under unloading or pathological conditions. Mechanistic studies confirm that elevated glycolysis and activation of the ERK1/2 and Wnt/*β*‐catenin pathways are the major contributors to the initiation of osteoinduction functions induced by osteocytes. Moreover, an osteocyte lysate‐based hydrogel is designed to establish a stockpile of “active osteocytes” to sustainably deliver bioactive proteins, resulting in accelerated healing through regulation of endogenous osteoblast/osteoclast homeostasis.

## Graphical abstract

This report describes a novel osteocyte lysate‐based hydrogel prepared from oscillating fluid flow (OFF)‐treated osteocytes. It can resist the effects of osteocytic highly dynamic properties and tumor cell attacks to induce a robust osteogenic response of osteoblasts and a suppressive effect on osteoclasts differetiation. The OFF‐treated osteocytes can manipulate osteogenesis/osteolysis balance through controlling the ERK1/2 and Wnt/*β*‐catenin pathways.

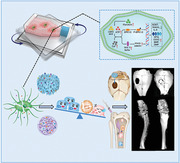

## 1 Introduction

The bone destruction caused by traumatic injury or osteolytic tumor cells often exceeds the regenerative ability of native bone and remains unhealed.^[^ ^1^, ^2^ ^]^ Recently, cell‐based therapies using in vivo transplantation for bone repair have become an attractive option.^[^ ^3^ ^]^ Osteocytes are believed to orchestrate bone remodeling in response to mechanical stimuli by transmitting soluble factors to effector cells (osteoblasts/osteoclasts).^[^ ^4^, ^5^, ^6^ ^]^ For instance, the direct sensing of bone strains might be sufficient to activate mechanosensitive osteocytes to exert osteoinductive effects in vivo.^[^ ^7^, ^8^ ^]^ Continuous cyclic mechanical stimulation of a mouse ulna resulted in increased expression of bone growth factor genes, such as dentin matrix protein 1 (DMP1), produced by osteocytes.^[^ ^9^ ^]^ However, this external mechanical stimulus can easily cause secondary damage to injured bone tissue. Additionally, the inaccessible location of osteocytes within the mineralized bone matrix also diminishes their sensitivity to perceived mechanical loading.^[^ ^10^ ^]^ Therefore, establishing an in vitro cell culture model that responds to mechanical loading would be a valuable approach to activate osteocytic osteoinductive function.

Osteocytes are sensitive to applied loading in the form of fluid flow stress, which in turn produces soluble factors to regulate surrounding osteoblast and osteoclast function in a paracrine manner.^[^ ^5^, ^11^, ^12^ ^]^ For instance, pulsating fluid flow (PFF) treatment^[^ ^13^ ^]^ and compressive cyclic force (CCF) treatment^[^ ^14^ ^]^ downregulate the expression of preosteoclast factors, such as sclerostin (SOST) and receptor activator for nuclear factor‐*κ* B ligand (RANKL), in vitro and increase the mRNA expression of the bone growth factor osteoprotegerin (OPG) in osteocytes, leading to inhibition of osteoclastogenesis, and an increase in osteoblast activity in 2D cell culture.^[^ ^15^ ^]^ However, since osteocytes are highly dynamic,^[^ ^16^, ^17^ ^]^ after removal of external stimulation, the gene expression gradually return to baseline levels within a few hours.^[^ ^14^, ^18^ ^]^ Therefore, even though pretreated osteocytes exhibit therapeutic potential after external stimulation, their in vivo application has been limited.

Moreover, pathologic factors produced by tumor cells in a diseased microenvironment inevitably affect osteocyte viability. The accumulation of damaged or apoptotic osteocytes surrounding the bone lesion area may send signals to promote osteoclast recruitment, local osteoclast formation, differentiation, and increased bone resorption.^[^ ^19^, ^20^ ^]^ Accordingly, osteocyte free supernatant has been developed to circumvent the abovementioned limitations. Hao et al. reported that PFF‐ and CCF‐induced osteocyte conditioned medium inhibited osteoclast formation and increased alkaline phosphatase (ALP) activity and osteoblast marker expression.^[^ ^12^, ^14^, ^15^ ^]^ However, due to the lower concentration of mediators released from pretreated osteocytes,^[^ ^14^ ^]^ osteoclastogenesis inhibition and osteogenesis promotion are observed to a lesser extent. Additionally, the need for highly specialized equipment has prevented the broad clinical application of osteocytes.^[^ ^12^, ^14^, ^15^ ^]^ Overall, for osteocyte‐based bone regeneration therapy, there is an unmet need to develop a facile and highly effective strategy to promote sufficient production of mediators in osteocytes to effectively regulate osteoblast and osteoclast function for an extended period of time and resist cellular instability.

**Scheme 1:**
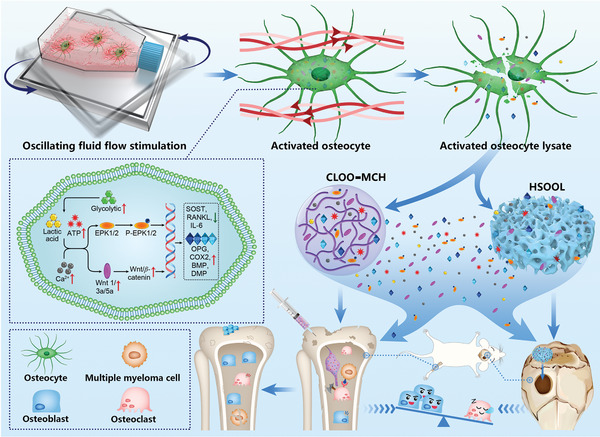
Schematic diagram of oscillating fluid flow activated osteocyte lysate‐based hydrogels for bone repair. The injectable methylcellulose hydrogel (CLOO‐MCH) and transplantable hydrogel scaffold (HSOOL) loaded with activated osteocyte lysates were prepared to regulate osteoblast/osteoclast activity at the bone injury site for bone repair and osteolysis treatment.

Recently, it has become widely accepted that in vivo interstitial fluid flow, a type of dynamic oscillating fluid flow, can activate the production of signaling molecules by osteocytes; these signaling molecules regulate the activity of the surrounding osteoblasts and osteoclasts.^[^ ^14^, ^15^, ^21^, ^22^ ^]^ Herein, we hypothesize that subjecting osteocytes to a controlled cyclic oscillating fluid flow in vitro can mimic the physiologically relevant mechanical signal that osteocytes experience in vivo, thus converting the osteocyte secretory phenotype and initiating the osteocytic bone anabolic response. Based on previous findings, cell lysates are rich in most paracrine proteins produced by living cells.^[^ ^23^ ^]^ They can be harnessed for tissue regeneration purposes in a fashion similar to their parent cells.^[^ ^24^ ^]^ In this study, we further confirmed that activated osteocyte lysates induced a robust and stable osteogenic response in osteoblasts and showed a suppressive effect on osteoclast differentiation. Furthermore, we systemically assessed the effect of our designed osteocyte lysate‐based hydrogels on the promotion of bone regeneration and prevention of bone resorption in both diseased and nonpathological environments (**Scheme** **1**). At a mechanistic level, we revealed the key roles of the ERK1/2 and Wnt/*β*‐catenin pathways during the OFF loading induced osteogenic response. Collectively, these investigations offered a promising approach to realize the feasibility of OFF‐mediated osteocyte mechanotransduction therapy to improve bone health and treat bone injury.

## 2 Results and Discussion

### 2.1 Oscillating Fluid Flow (OFF) as a Stimulus for Initiating the Bone Anabolic Response in Osteocytes

**Figure 1:**
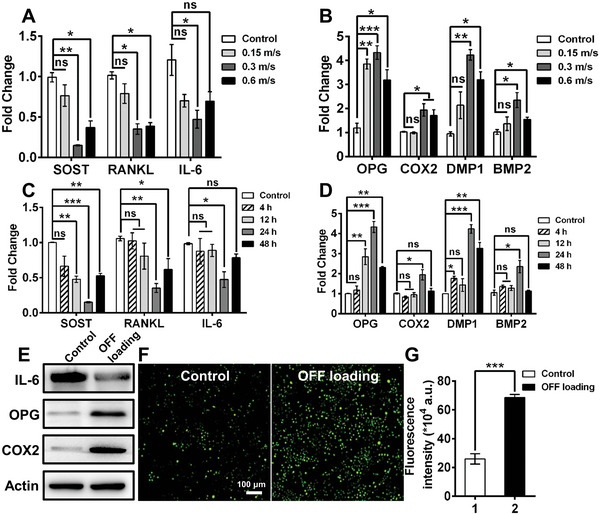
Osteogenic induction ability of osteocytes with different OFF loading treatments. A) mRNA expression levels of pro‐osteoclast factors and B) bone growth factors in osteocytes with OFF loading at C) different flow velocities and D) different stimulation times. E) Western blotting analysis of the protein expression levels in osteocytes after OFF loading treatment (0.3 m s^−1^, 24 h). F) Representative images of Fluo‐4 AM staining indicating Ca^2+^ (green) in osteocytes by OFF loading treatment (0.3 m s^−1^, 24 h). G) The intracellular Ca^2+^ concentration was estimated by determining the average fluorescence intensity per unit area using ImageJ software. The data are presented as the mean ± SEM (*n* = 3 independent experiments, each with 3 technical replicates). Significance is indicated as *p < 0.05, **p < 0.01, and ***p < 0.001; ns, not significant.

In vivo, the maximum steady state velocities of interstitial fluid (a type of dynamic OFF) through the bone matrix and cortical canals are 0.04–0.2 and 0.6–0.9 m s^−1^, respectively.^[^ ^25^ ^]^ When the fluid flow velocity is in this range, the osteocytic osteogenesis process will be activated due to corresponding mechanical loading.^[^ ^26^ ^]^ Conversely, extra mechanical loading resulting from high fluid flow in the supraphysiological range induce osteocyte‐mediated osteoclastogenesis.^[^ ^27^ ^]^ Therefore, in this study, OFF was utilized because it is similar to the in vivo forces experienced by osteocytes.^[^ ^22^ ^]^ To verify whether OFF loading can induce an osteocytic bone anabolic response similar to that induced by interstitial fluid flow, we subjected MLO‐Y4 osteocytes to constant OFF stimulation at different fluid flow velocities (0.15, 0.3, and 0.6 m s^−1^) and for different stimulation times (4, 12, 24, and 48 h). As shown in **Figure** **1**A, compared to treatments at flow rates of 0.15 and 0.6 m s^−1^, the lowest expression levels of pro‐osteoclast factors (SOST, RANKL, and interleukin (IL‐6)) in osteocytes were found at a flow rate of 0.3 m s^−1^. Conversely, the expression of OPG, DMP1, cyclooxygenase‐2 (COX2), and bone morphogenetic protein 2 (BMP2), which are related to osteocyte‐mediated bone formation in MLO‐Y4 cells, was considerably increased after OFF loading treatment for 24 h (Figure 1B). Moreover, we compared the 0.3 m s^−1^ * 24 h group with the 0.15 m s^−1^ * 48 h and 0.6 m s^−1^ * 12 h groups. Compared to the 0.3 m s^−1^ * 24 h group, the 0.6 m s^−1^ * 12 h group showed a similar inhibitory effect on the expression of the pro‐osteoclast factors SOST and IL‐6, but these conditions did not effectively promote bone growth factor expression. For the 0.15 m s^−1^ * 48 h group, only the osteogenic factor COX2 showed a nearly fourfold increase in expression, but no inhibitory action on pro‐osteoclast factor production was observed compared to the other groups (Figure S1, Supporting Information). Interestingly, we found that the total protein content in osteocytes was higher in the higher flow velocity group (0.3–0.6 m s^−1^) than in the lower flow velocity group (0–0.15 m s^−1^) (Figure S2, Supporting Information). Based on these data, 0.3 m s^−1^ was utilized as the optimized flow rate in subsequent experiments. Furthermore, we detected the osteocyte response to OFF loading after different durations of stimulation. Figure 1C shows that the expression of pro‐osteoclast factors (IL‐6, SOST, and RANKL) after 24 h of treatment was much lower than that in the other groups. Figure 1D also indicates that the expression of osteogenic factors (OPG, COX2, DMP1, and BMP2) was significantly increased under 24 h of OFF loading compared with the other stimulation times. The expression levels of the IL‐6, OPG, and COX2 proteins within osteocytes were further examined through western blotting assays. As shown in Figure 1E, OFF loading blocked IL‐6 protein expression and increased OPG and COX2 proteins production in osteocytes compared with unloading group, which was consistent with the above mRNA expression results. Additionally, intracellular calcium mobilization is an important early response to mechanical loading, and calcium release is also an essential prerequisite for activating a multitude of signaling pathways in osteocytes.^[^ ^28^, ^29^ ^]^ Therefore, we examined the concentration of free calcium ions (Ca^2+^) in osteocytes. Compared to the control group, a considerably higher fluorescence intensity of the calcium probe was detected in MLO‐Y4 cells in the OFF loading group (Figure 1F,G), suggesting that the Ca^2+^ signals in osteocytes increased with OFF loading. These results indicated that an OFF velocity of 0.3 m s^−1^ and a stimulation duration time of 24 h provided the strongest osteogenic cue in osteocytes.

### 2.2 OFF‐Treated Osteocyte Lysates Modulate Osteoblast and Osteoclast Differentiation in Diseased and Nonpathological Environments

**Figure 2:**
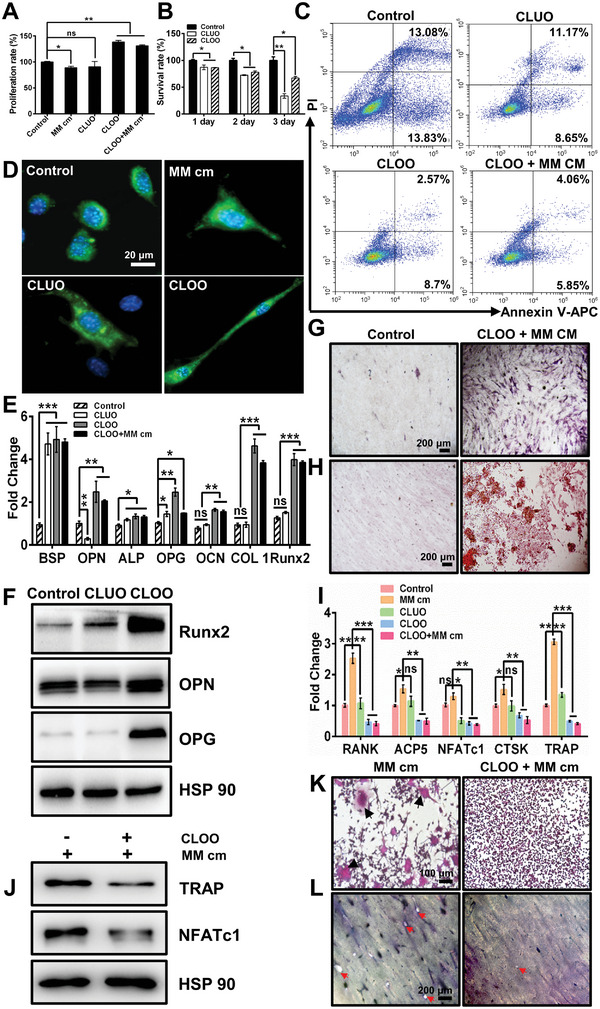
The effect of CLOO on the biological behavior of osteoblasts and osteoclasts. MC3T3‐E1 were co‐cultured with different stimuli for 1–14 days. A) The viability of MC3T3‐E1 cells cultured under different conditions for 24 h. B) Survival rate of 5TGM1 cells after treatment with CLOO and CLUO at days 1–3. C) Flow cytometry analysis of MC3T3‐E1 cell apoptosis caused by physical communication between osteoblasts and myeloma cells in the presence of CLOO, CLUO, and CLOO+MMcm for a period of 4 days. The cells were stained with Annexin V‐APC and PI for analysis. D) Representative images of changes in MC3T3‐E1 cell morphology under different stimulation conditions on day 1; plasma membrane staining with DIO (green) and nuclear staining with DAPI (blue). Scale bar: 50 µm. The expression of key osteogenic markers in MC3T3‐E1 cells was assessed on day 7 via E) qRT‐PCR and F) western blotting. G) ALP staining and H) Alizarin red staining were performed to visualize the effect of CLOO on osteogenic differentiation under MM cm condition on days 7 and 14, respectively. RAW 264.7 were co‐cultured with different stimuli for 5–7 days. I) qRT‐PCR and J) western blotting evaluation of osteoclast function effector gene and protein expression levels in RAW 264.7 on day 5. K) TRAP staining and L) bone resorption assays were performed to analyze osteoclast formation and activity under MM cm condition after coculturing with CLOO on day 7 (black arrow indicates positive multinucleated osteoclasts; red arrow indicates etched bone defects). The data represent the mean ± SEM for *n* = 3 independent experiments, each with 3 technical replicates. Significance is indicated as *p < 0.05, **p < 0.01, and ***p < 0.001; ns, not significant.

Osteocytes are highly dynamic, and their regulatory function is transient as mechanical stimuli cease.^[^ ^16^ ^]^ As shown in Figure S3, Supporting Information, when the mechanical stimuli (OFF loading) were removed for 24 h, the expression levels of OPG, DMP1, and COX2 in the osteocytes treated with OFF loading decreased rapidly and reached the expression level in untreated osteocytes. Similarly, the decrease in the expression of pro‐osteoclast factors (IL‐6, SOST, and RANKL) after OFF treatment was lost over time after cessation of flow and reached or exceeded baseline levels. These results indicated that the bone anabolic responses of osteocytes depended on continuous external OFF loading stimuli, which hindered the implementation of osteocytes for in vivo application. Cell lysates from osteocytes subjected to OFF were enriched in most of the secreted active molecules and were more easily preserved than cells.^[^ ^23^ ^]^ Therefore, we hypothesized that cell lysates derived from OFF‐treated osteocytes (CLOO) could be harnessed for bone regenerative purposes in a fashion similar to OFF‐treated osteocytes. To verify the capacity of CLOO to regulate osteogenic potency in both diseased and nonpathological environments, we used multiple myeloma conditioned medium (MM cm) to mimic diseased environments. First, we detected the proliferation of preosteoblast (MC3T3‐E1) cells after coculturing with different stimuli. As shown in **Figure** **2**A, CLOO exhibited a significant promoting effect on MC3T3‐E1 cell proliferation compared to cell lysates derived from untreated osteocytes (CLUO), regardless of whether a diseased environment was present. We also found that both CLOO and CLUO displayed a clear inhibitory effect on tumor growth, as shown in Figure 2B. The viability of 5TGM1 cells was restricted and appeared to decrease with prolonged treatment with CLOO and CLUO. Consistent with the effect observed in previous studies, osteocytes can suppress cancer cell growth through cytokine secretion.^[^ ^30^ ^]^ This result indicated that osteocyte lysates possessed a function similar to that of intact osteocytes. Notably, the pathologic factors produced by myeloma cells have been demonstrated to destroy the viability and cellular function of osteocytes and induce osteoblast apoptosis.^[^ ^19^, ^20^ ^]^ To demonstrate that the inhibitory effect of CLOO on MC3T3‐E1 cell apoptosis was not affected by tumor cells, a flow cytometer assay was applied. As shown in Figure 2C, after the osteoblasts were cocultured with 5TGM1 cells and CLOO for 4 days, the apoptosis rate of MC3T3‐E1 cells (≈9.91%) was significantly lower than that of cells in the Control group (26.91%) and CLUO group (19.82%). Subsequently, to detect CLOO‐induced osteoblast migration, the number of MC3T3‐E1 cells passing through a Transwell membrane was counted. On day 1 and day 2, the average number of cells per field was much higher in the CLOO group than in the CLUO group (Figure S4, Supporting Information).

To analyze the effects of CLOO on the osteogenic differentiation of MC3T3‐E1 cells, we probed preosteoblasts for changes in morphology and primary osteogenic gene expression. Osteoblasts can be embedded in bone matrix and differentiate into osteocytes.^[^ ^16^ ^]^ As shown in Figure 2D, we found that the morphology of MC3T3‐E1 cells changed dramatically after treatment with CLOO for 1 day; the cells transitioned from plump polygonal preosteoblasts to stellate, longer dendritic osteocytes. Moreover, the activity of the early osteogenic marker ALP in the CLOO‐treated group increased with increasing CLOO dosage (50, 100, and 300 µg mL^−1^) (Figure S5, Supporting Information). Compared with the CLUO group, treatment with CLOO also led to increased expression of primary osteogenic genes (bone sialoprotein (BSP), osteopontin (OPN), ALP, OPG, osteocalcin (OCN), collagen type1 (COL1), and runt‐related transcription factor 2 (Runx2)) and related proteins (Runx2, OPN, and OPG) in MC3T3‐E1 cells (Figure 2E,F). Additionally, elevated ALP production and enhanced mineralized nodule formation were observed in the CLOO treatment group compared with the other treatment groups (Figure 2G,H; Figures S6,S7, Supporting Information). These results suggested that CLOO was capable of inducing further differentiation and maturation of preosteoblasts without being influenced by the diseased environment in vitro.

To further probe the effect of CLOO on osteoclast differentiation and formation, we cultured RAW 264.7 cells in the presence of CLOO and RANKL. The expression of osteoclast‐specific functional effector genes (cathepsin K (CTSK), RANK, nuclear factor of activated T‐cells, cytoplasmic 1 (NFATc1), andtartrate‐resistant acid phosphatase (TRAP)) and the corresponding proteins (TRAP and NFATc1) was evaluated via qPCR and western blotting analysis. As shown in Figure 2I, the expression of the abovementioned genes was significantly downregulated in the CLOO and CLOO+MM cm groups compared with the free MM cm stimulation group. Meanwhile, a similar inhibitory effect on osteoclastic gene expression levels was observed in the CLUO treatment group, but to a lesser extent. Furthermore, lower TRAP and NFATc1 protein expression levels were detected in the CLOO+MM cm group than in the free MM cm group (Figure 2J). As shown in Figure S8, Supporting Information, compared to the control group, MM cm treatment amplified osteoclast formation, as evidenced by the increased number of positive multinucleated osteoclasts (black arrow). This finding was consistent with those of previous studies on myeloma‐induced osteoclast activation. However, a dramatic decrease in TRAP‐positive multinucleated osteoclasts was observed in the CLOO+MM cm‐treated group (Figure 2K). This finding indicated that CLOO could alleviate these effects caused by myeloma cells. Additionally, no suppression of osteoclast formation was observed in the CLUO group (Figure S8, Supporting Information). When osteoclasts were added onto a bovine bone surface for 7 days, a significant reduction in the amount and area of bone resorption was observed with CLOO treatment. However, more etching bone defects (red arrow) and inorganic mineral loss (deep staining) occurred in the free MM cm group and CLUO group (Figure 2L; Figure S9, Supporting Information). These results confirmed that CLOO had an inhibitory effect on bone resorption in vitro with or without MM cm stimulation.

### 2.3 Crosstalk between OFF Stimuli and Osteocytic Molecular Signaling Pathways

**Figure 3:**
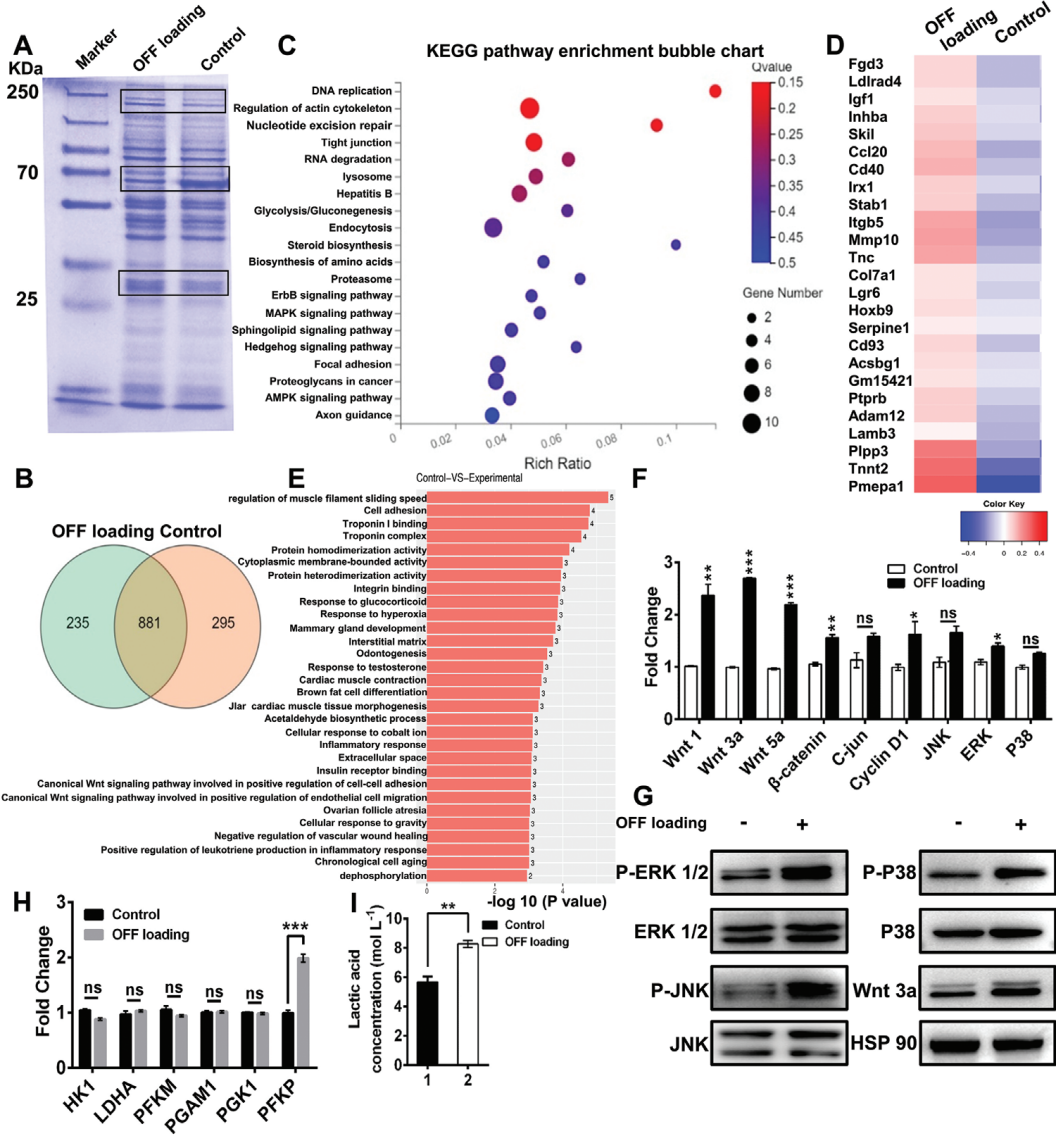
Mechanistic analysis of osteocyte (MLO‐Y4)‐induced regulation of osteoclasts/osteoblasts in response to OFF loading. A) Coomassie staining of total proteins in OFF loading osteocytes separated by SDS–PAGE. The rectangular area identifies the proteins with differential expression levels between the OFF loading and unloading groups. B) Venn diagrams displaying the number of proteins identified in osteocyte cultured in control condition and in OFF loading condition. C) KEGG pathway enrichment analysis and prediction of the function of specifically expressed proteins in osteocytes with OFF loading treatment. D) A heatmap was generated to assess the mRNA expression levels in MLO‐Y4 cells under different culture conditions. Red and blue denote high and low expression, respectively. E) GO enrichment analysis and prediction of the functions of specific genes expressed in osteocytes with or without OFF loading treatment. F) qRT‐PCR and G) western blotting assays analysis of activation of the MAPK and Wnt/*β*‐catenin pathways in osteocytes with or without OFF loading. H) Expression of glycolysis pathway‐related genes in MLO‐Y4 cells before and after OFF loading stimulation. I) Lactic acid level in MLO‐Y4 cells before and after OFF loading stimulation. The data are presented as the mean ± SEM for *n* = 3 independent experiments, each with 3 technical replicates. *p < 0.05, **p < 0.01, and ***p < 0.001; ns, not significant.

Extensive evidence has revealed that osteocytes are able to discern different forms of mechanical stimulation and various stimuli applied at different frequencies, thereby adjusting distinct signaling pathways.^[^ ^4^, ^16^, ^31^, ^32^ ^]^ For example, pulsating fluid flow loading activates the parathyroid hormone and INOS signaling pathways.^[^ ^30^ ^]^ To elucidate the signal transduction pathway involved in the osteoinduction process under OFF loading, we screened the differentially expressed proteins in osteocytes with and without OFF loading through SDS–PAGE analysis (**Figure** **3**A). According to liquid chromatography‒tandem mass spectrometry (LC‒MS/MS) results, approximately 235 specific proteins were identified in osteocytes subjected to OFF, and 295 specific proteins were identified in untreated osteocytes (Figure 3B). As shown in Figure 3C and Figures S10 and S11, Supporting Information, KEGG pathway analysis indicated that a considerable number of specific proteins in osteocytes subjected to OFF were involved in the classic MAPK signaling pathway (including ERK1/2, P38, and JNK). Moreover, high expression of the mechanosensitive protein tight junction protein 1 (TJP1) was also found in OFF‐treated osteocytes (Figure S12, Supporting Information). Meanwhile, we further investigated the mRNA expression profile by conducting mRNA sequencing assay (mRNA‐seq). Figure 3D shows a cluster of genes that were upregulated in osteocytes subjected to OFF loading compared to untreated osteocytes. Interestingly, we found that the expression of Wnt/*β*‐catenin pathway‐related genes (Lgr6, Hoxb9, and Plpp3), cell adhesion‐related proteins (lamb3, Adma12, Ptprb, Col7a1, stab1, and CD40) and extracellular matrix (ECM)‐related proteins (Tnc and Itgb5) was upregulated in OFF‐treated osteocytes compared to untreated osteocytes (Figure 3D,E). These results indicated that the OFF stimulus induced changes in osteocyte adhesion behavior and activated the Wnt/*β*‐catenin pathway. The enhanced Tnc and Itgb5 expression suggested that the ECM signaling pathway might be associated with cellular mechanotransduction. Subsequently, qRT‐PCR and western blotting analyses were performed to confirm the LC‒MS/MS and mRNA‐seq‐derived predictions regarding signaling pathways. Consistent with the predictions, clear upregulation of the mRNA expression levels of Wnt family proteins (Wnt1, Wnt3a, and Wnt5a) and Wnt target genes (*β*‐catenin and cyclin D1) were observed in OFF‐treated osteocytes (Figure 3F). Moreover, considerably elevated Wnt3a protein expression was detected in OFF‐treated osteocytes (Figure 3G), implying that OFF loading might lead to activation of the Wnt/*β*‐catenin pathway in osteocytes. With respect to the MAPK signaling pathway, OFF loading did not trigger JNK and P38 gene expression (Figure 3F). However, significantly increased ERK1/2, JNK, and p38 phosphorylation levels were observed in osteocytes under OFF loading conditions (Figure 3G; Figure S13, Supporting Information). In addition, mass spectrometry analysis showed that glycolysis was more dominant than the tricarboxylic acid cycle (TCA) in osteocytes subjected to OFF loading. The identified proteins in OFF‐treated osteocytes were specifically related to the glycolysis pathway (Figure 3C; Figure S14, Supporting Information). In contrast, the specific proteins identified in untreated osteocytes indicated that the cells mainly depended on the TCA cycle to support energy demand (Figures S15,S16, Supporting Information). We further analyzed the important enzymes involved in the glycolysis pathway and the end‐product of glycolysis. Figure 3H,I, shows that the expression of phosphofructokinase (PFKP) and the level of lactic acid were significantly upregulated in OFF‐treated osteocytes compared to untreated osteocytes. This phenomenon was attributed mainly to the increase in energy produced by the glycolysis pathway, which could support the activation of multiple signaling pathways in osteocytes. Collectively, OFF‐treated osteocytes showed marked signaling pathway activation, transcription and protein‐level responses, which might induce osteocytes to regulate osteoblast/osteoclast activity.

### 2.4 Effects of the MAPK Pathway and Wnt/*β*‐Catenin Pathway on Osteocyte‐Induced Bone Remodeling

**Figure 4:**
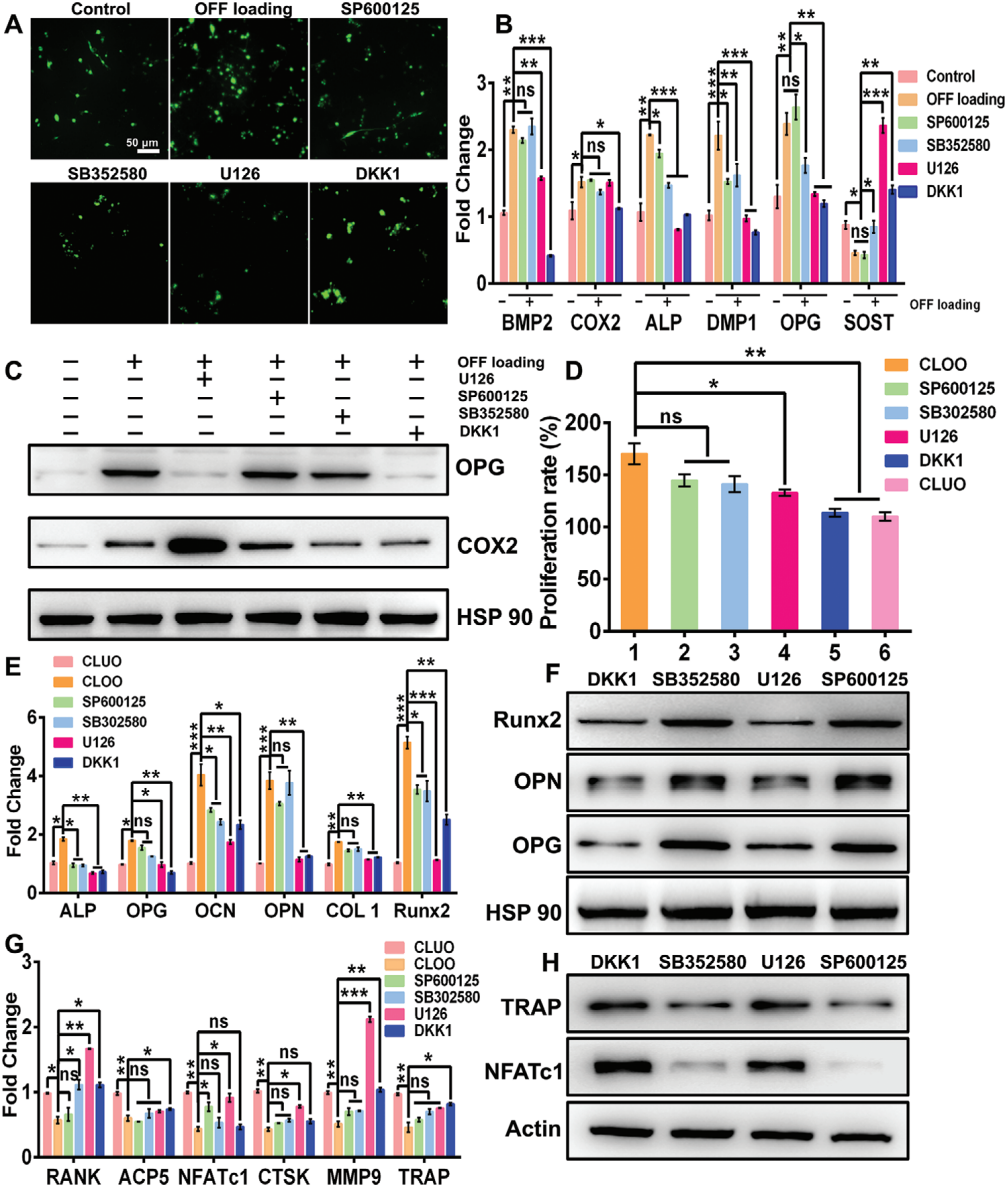
The ERK1/2 and Wnt/*β*‐catenin pathways were the major regulators of the bone anabolic effect of osteocytes in response to OFF loading. A) Intracellular Ca^2+^ levels before and after inhibitor (SP600125, SB352580, U126, and DKK1) treatment under OFF loading stimulation. B) qRT‐PCR and C) western blotting assays to detect bone metabolism‐related gene expression in inhibitor pre‐treated osteocytes with OFF loading stimulation. D) Promoting effect on the proliferation rate of MC3T3‐E1 cells induced by CLOO and various inhibitor pretreated CLOO on day 1. E) qRT‐PCR and F) western blotting assays were conducted to evaluate the suppressive effect of inhibitors on osteogenic marker expression in MC3T3‐E1 cells induced by various inhibitor pretreated CLOO on day 7. G,H) Expression level of osteoclast markers in RAW 264.7 cells after coculturing with CLOO and various inhibitor pretreated CLOO for 5 days. The data are presented as the mean ± SEM for *n* = 3 independent experiments, each with 3 replicates. *p < 0.05, **p < 0.01, and ***p < 0.001; ns, not significant.

To demonstrate that the MAPK and Wnt/*β*‐catenin pathways were the major contributors to osteocyte‐mediated bone remodeling under OFF loading conditions, an inhibitor assay was performed. **Figure** **4**A shows that the inner calcium concentration in inhibitor‐treated osteocytes was downregulated significantly under OFF loading stimulation. Moreover, compared to that in osteocytes treated with JNK and p38 inhibitors (SP600125 and SB352580), the expression of bone growth factors (OPG, DMP1, and BMP2) was dramatically decreased in OFF‐treated osteocytes after Wnt/*β*‐catenin and ERK1/2 inhibitor (dickkopf‐1(DKK1) and U126) treatments (Figure 4B). Conversely, the expression of the osteoclast factor SOST was upregulated in OFF‐treated osteocytes after DKK1 and U126 treatments (Figure 4B). Figure 4C shows that OPG protein expression was obviously decreased in the DKK1 and U126 treatment groups. However, COX2 protein expression did not show a similar tendency. COX2 is a key enzyme that helps synthesize prostaglandin, which has been reported to enhance new bone formation in response to mechanical stimuli.^[^ ^10^ ^]^ Thus, the MAPK pathway and Wnt/*β*‐catenin pathway might have no effect on hormone secretion by OFF‐treated osteocytes. Furthermore, cell lysates from inhibitor‐treated osteocytes were utilized to stimulate MC3T3‐E1 cells. As shown in Figure 4D and Figure S17, Supporting Information, when the ERK1/2 and Wnt/*β*‐catenin signaling pathways were blocked using U126 and DKK1 inhibitors, respectively, the enhancement of osteoblast proliferation induced by CLOO treatment was significantly reduced on day 1 and day 3. However, JNK and P38 inhibitors could not reverse the increase in osteoblast growth. In addition, we found that DKK1 and U126 inhibitors restricted osteoblast differentiation and maturation through CLOO mediation. As shown in Figure 4E,F, in preosteoblasts (MC3T3‐E1 cells), osteogenic gene expression (ALP, OPG, OCN, OPN, COL1, and Runx2) and the expression of related proteins (OPG, OPN, and Runx2) were dramatically decreased in the U126 and DKK1 inhibitor groups compared with the JNK and P38 inhibitor groups. As shown in Figure S18, Supporting Information, the reduced ALP staining and Alizarin red staining revealed that the DKK1 and U126 inhibitors induced osteogenesis suppression, suggesting that silencing of the ERK1/2 and Wnt/*β*‐catenin signaling pathways could reduce the positive regulatory effect of osteocytes on osteogenesis. To further verify the effect of related signaling pathways in osteocytes on osteoclast activity, we blocked the MAPK and Wnt/*β*‐catenin signaling pathways in OFF‐treated osteocytes and then assessed the effect of osteocyte lysates on the expression of osteoclast‐specific functional genes (RANK, acid phosphatase 5 (Acp5), NFATc1, CTSK, matrix metalloprotein 9 (MMP9), and TARP) and the corresponding proteins (TRAP and NFATc1). Compared to p38 and JNK inhibitors, DKK1 and U126 inhibitors eliminated the restriction of osteoclast‐specific functional gene expression induced by CLOO treatment (Figure 4G). Figure 4H shows a distinct increase in the TRAP and NFATc1 levels in the DKK1 and U126 inhibitor groups compared with the p38 and JNK inhibitor groups. Furthermore, an increase in the number of TRAP‐positive multinucleated giant osteoclasts was observed in the U126 and DKK1 inhibitor groups compared with the other inhibitor groups (Figure S19, Supporting Information). Consistent with the TRAP staining results, CLOO no longer possessed the ability to deplete osteoclasts after the Wnt/*β*‐catenin pathway and ERK1/2 pathway were inhibited in osteocytes, as shown by the increase in etching bone defects in the U126 and DKK1 groups (Figure S19, Supporting Information). In contrast, p38 and JNK inhibitors did not affect osteocyte function in inhibiting bone resorption. Overall, the ERK1/2 and Wnt/*β*‐catenin pathways played key roles in pro‐osteogenic differentiation and the anti‐osteoclast formation/activation response to OFF‐treated osteocytes.

### 2.5 An Injectable Methylcellulose Hydrogel Loaded with CLOO (CLOO‐MCH) Was Designed to Regulate Osteoblast/Osteoclast Differentiation

**Figure 5:**
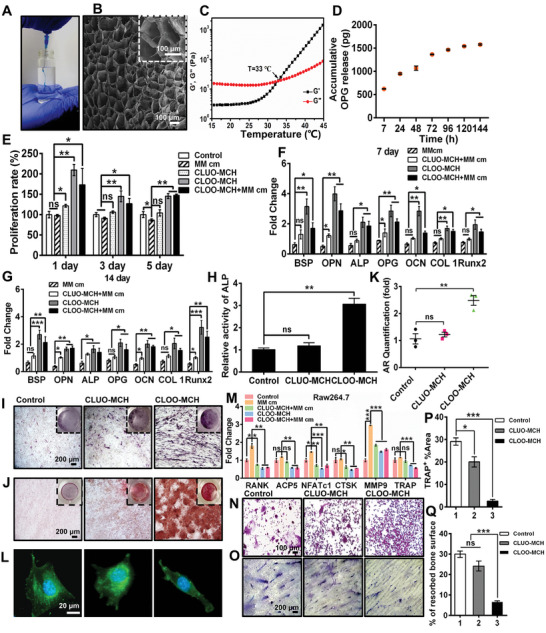
An injectable CLOO‐MCH was developed to induce osteogenesis and inhibit osteoclasts. A) Photograph of injection of CLOO‐MCH solution in water at 35 °C. B) A freeze‐dried CLOO‐MCH showed its pore structure by scanning electron micrographs. C) Rheological data from temperature ramp scanning analyses. D) Accumulative OPG release profiles from CLOO‐MCH. An injectable osteocyte lysate‐based hydrogel was developed to deliver osteocyte lysates. The regulatory function of the hydrogel was analyzed in an in vitro coculturing system. E) The viability of MC3T3‐E1 cells after incubation with different stimuli for 1 day, 3 days, and 5 days. F,G) Osteogenic gene expression in MC3T3‐E1 cells after coculturing with different stimuli for 7 days and 14 days. H) ALP activity in MC3T3‐E1 cells on day 1 of coculture. I) ALP staining on day 7 of coculture, J,K) Alizarin red staining and quantification on day 14 of coculture. The inset shows images of the entire stained well. L) A morphological analysis was performed to visualize the effect of CLOO‐MCH on osteogenic differentiation after 2 days of coculture. M) qRT‐PCR analysis of osteoclast function effector gene expression in RAW264.7 cells on day 5 of coculture. N,P) Representative images of TRAP staining and quantitative analysis results after 7 days of coculture. O,Q) A bone resorption assay and quantitative analysis of the bone resorption surface were performed to assess osteoclast differentiation after 7 days of coculture. The data represent the mean ± SEM for *n* = 3 independent experiments, each with 3 technical replicates. *p < 0.05, **p < 0.01, and ***p < 0.001; ns, not significant.

Multiple myeloma bone disease (MBD) is characterized by massive osteolysis that occurs near myeloma cells in the medullary cavity.^[^ ^33^, ^34^, ^35^ ^]^ We prepared an injectable temperature‐sensitive methylcellulose hydrogel as a carrier to encapsulate and release CLOO for MBD therapy (CLOO‐MCH). As shown in **Figure** **5**A, fast gelation occurred with CLOO‐MCH when the temperature increased. The microporous structure and pore size of CLOO‐MCH were observed by scanning electron microscopy (SEM). Figure 5B shows that CLOO‐MCH had a well‐connected porous structure, and the pore size was ≈80 µm. The gelation temperature of CLOO‐MCH was measured using a rheometer (Figure 5C). When the temperature rose to 33 °C, the storage modulus *G*′ was equal to the loss modulus *G*″, indicating that 33 °C was the gelation temperature of CLOO‐MCH. Figure S20, Supporting Information, shows that 58.3% of the total protein (350 µg mL^−1^) was released from CLOO‐MCH after 6 days. The osteocyte lysates promoted the proliferation of osteoblasts, as the total protein concentration was over 86 µg mL^−1^ (Figure S21, Supporting Information). Therefore, these results demonstrated that CLOO‐MCH possessed promising biological activity. OPG is usually regarded as the critical regulatory factor that orchestrates bone metabolic activity in response to mechanical stimuli.^[^ ^36^ ^]^ Figure 5D shows that CLOO‐MCH exhibited sustained release of OPG, with the amount reaching 1.6 ng on day 6, thus achieving the effective concentration.^[^ ^37^ ^]^ Furthermore, when osteoblasts were cultured with CLOO‐MCH under MM cm stimulation, the osteoblast proliferation rate in the CLOO‐MCH group was significantly increased on days 1, 3, and 5, consistent with the effect of osteocyte lysates in vitro (Figure 5E). Moreover, compared to the control group, the CLOO‐MCH group showed increased osteogenic gene expression (BSP, OPN, ALP, OPG, OCN, COL1, and Runx2) and elevated ALP activity and mineralization, further indicating the enhanced osteogenesis capacity of CLOO‐MCH (Figure 5F–K). Additionally, when cultured with CLOO‐MCH, osteoblasts exhibited a typical slender morphology, which indicated an increase in osteoblast differentiation (Figure 5L). In terms of osteoclast biological function, as shown in Figure 5M, the expression of osteoclast function effector genes (RANK, ACP5, NFATc1, MMP9, and TRAP) was reduced by coculturing with CLOO‐MCH. Compared to the control group, a clear decrease in the area of TRAP‐positive cells and bone resorption was detected in the CLOO‐MCH group (Figure 5N–Q). However, there was no significant difference between the CLUO‐MCH group and the control group. The results confirmed that CLOO‐MCH exerted a bifunctional effect similar to that of CLOO on pro‐osteogenic differentiation and anti‐osteoclast activation.

### 2.6 CLOO‐MCH Showed a Superior Therapeutic Effect in a Multiple Myeloma Bone Disease (MBD) Mouse Model

**Figure 6:**
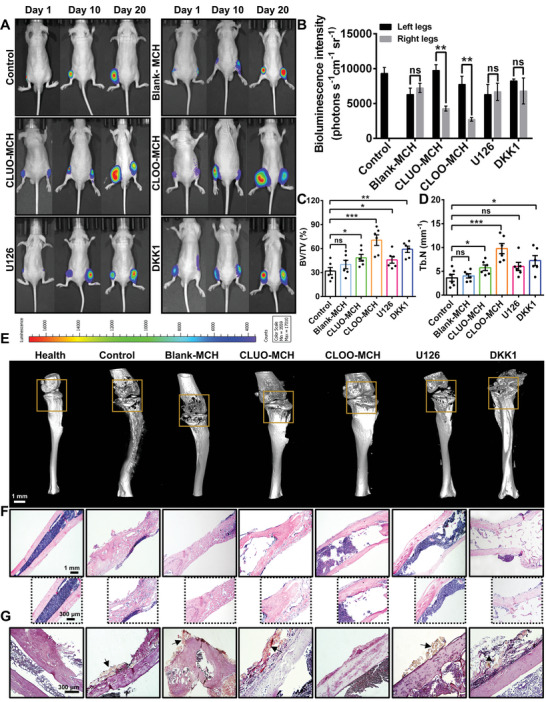
CLOO‐MCH exhibited clear prevention and suppression of massive osteolysis induced by 5TGM1 cells. A) On day 1, 5TGM1‐luc cells were injected into the tibial of the mice. On day 2, different types of hydrogels were injected into the tibial of the mice. Bioluminescence images of the tibial tissue at different time points post‐injection with 5TGM1‐luc cells and different types of hydrogels. B) Quantitative analysis of the bioluminescence intensity of 5TGM1‐luc cells in tibial tissue after 20 days of post‐injection in mice (*n* = 6 animals per group). C) Quantitative analyses of the BV/TV ratio and D) Tb.N in tibiae collected from different groups after 20 days of post‐injection with 5TGM1‐luc cells and different types of hydrogels in mice. The bar represents the mean ± SEM for *n* = 6 replicates per group. *p < 0.05, **p < 0.01, and ***p < 0.001; ns, not significant. E) Representative 3D reconstructed images of the tibiae collected from different groups after 20 days of post‐injection with 5TGM1‐luc cells and different types of hydrogels in mice. F) H&E and G) TRAP staining of tibial sections collected from mice in the indicated group after 20 days of post‐injection with 5TGM1‐luc cells and different types of hydrogels in mice.

An MBD mouse model was utilized to evaluate the inhibitory effect of CLOO‐MCH on myeloma cell (5TGM1)‐induced bone damage. On day 1, both the left and right tibias of mice were injected with 5TGM1‐luc cells. The untreated right tibias in the control group were set as the healthy group, and the left tibias treated with free 5TGM1‐luc cells were set as the negative control group. On day 2, the mice in the experimental group were randomly divided into five groups. The right tibias of the mice that were pre‐inoculated with myeloma cells were injected with blank MCH, CLUO‐MCH, CLOO‐MCH, anti‐ERK1/2 CLOO‐MCH (U126), or anti‐Wnt/*β*‐catenin CLOO‐MCH (DKK1). Bioluminescence imaging was utilized for tumor monitoring on days 1, 10, and 20. As displayed in **Figure** **6**A,B, the bioluminescence signal in the left tibia increased rapidly in all groups. However, tumor growth in the right tibias of mice treated with CLOO‐MCH and CLUO‐MCH was partially inhibited compared with that in the blank MCH and U126 groups (Figure 6A,B; Figure S22, Supporting Information). This result was consistent with the above in vitro studies, indicating that the osteocyte lysates inhibited the proliferation of 5TGM1 cells. Furthermore, micro‐CT analysis demonstrated that the ratio of bone volume to tissue volume (BV/TV) and trabecular number (Tb.N) values for the right tibia diaphysis in the CLOO‐MCH group were much higher than those in the control group and blank MCH group (Figure 6C,D). Interestingly, the bone preservation efficacy of CLOO‐MCH was partially inhibited when the activation of the ERK1/2 and Wnt/*β*‐catenin pathways was blocked in osteocytes. According to the 3D reconstructed threshold‐based CT images (Figure 6E; Figure S22, Supporting Information), the severity of bone destruction at the joint site (in the orange box) was reduced significantly in the CLOO‐MCH group compared with the blank MCH group and the control group, while CLOO‐MCH pretreated with U126 and DKK1 did not achieve a satisfactory effect in preventing osteolysis. Consistent with this, histological analyses, including hematoxylin and eosin (H&E) staining, demonstrated that the bone volume and bone surface were both well maintained in the CLOO‐MCH group (Figure 6F). In contrast, mottling and patchy, caved or faveolated bone destruction caused by myeloma cells were evident in the control group and blank MCH group (Figure S23, Supporting Information). Notably, compared to the effect of CLOO‐MCH, complete remission of this lytic bone destruction could not be achieved using anti‐ERK1/2 CLOO‐MCH or anti‐Wnt/*β*‐catenin CLOO‐MCH (Figure 6E). Additionally, TRAP staining suggested that markedly fewer osteoclasts were present in the CLOO‐MCH group than in the other groups (except for the healthy group) (Figure 6G). Moreover, treatment with anti‐ERK1/2 CLOO‐MCH or anti‐Wnt/*β*‐catenin CLOO‐MCH led to an increase in the number of osteoclasts (TRAP‐positive cells) compared with CLOO‐MCH treatment. Overall, these results showed that CLOO‐MCH could provide a promising strategy for the treatment of tumor‐induced bone destruction and unbalanced bone homeostasis.

### 2.7 An OFF‐Treated Osteocyte Lysate‐Based Hydrogel Scaffold (HSOOL) Promoted Calvarial Bone Healing

**Figure 7:**
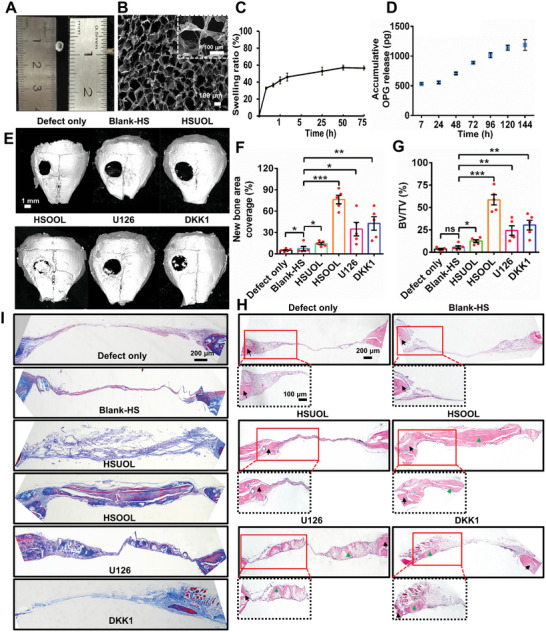
In vivo evaluation of HSOOL function in the repair of critical‐sized bone defects. A) Photograph of HSOOL. B) SEM images of a freeze‐dried HSOOL. C) Swelling ratio of HSOOL. D) Accumulative OPG release profiles of HSOOL. The data represent the mean ± SEM for *n* = 3 independent experiments, each with 3 technical replicates. 3 mm defects were made in the parietal bone in nude mice, a thin layer of osteocyte lysate‐based hydrogel scaffold was overlaid on the defect and secured. E) Representative micro‐CT images of mouse calvarial bone regeneration after 8 weeks of transplantation with different hydrogel scaffolds. F) Quantification of the new bone area coverage over the defect site, as imaged by CT at 8 weeks post‐procedure. G) BV/TV ratio. The bar represents the mean ± SEM (*n* = 5 animals per group). *p < 0.05, **p < 0.1, and ***p < 0.001; ns, not significant. H) Representative H&E staining and I) Masson trichrome staining images of calvarial bone with transplantation of different hydrogel scaffolds at 8 weeks. The dashed boxes (black) show high‐magnification images of the specified region in the upper image. Black arrowheads indicate native bone tissue, and green arrowheads indicate new bone tissue.

To investigate the effect of osteocyte lysates on critical‐sized bone defect repair, we fabricated a calvarial bone defect model with 3 mm defects using BALB/c nude mice (Figure S24, Supporting Information). Because the regeneration of calvarial bone requires long‐term treatment, we prepared a hydrogel scaffold loaded with OFF‐treated osteocyte lysates (HSOOL). The scaffold had a slow degradation rate to achieve sustained release of bioactive molecules and facilitate augmentation of the innate regeneration ability of bone tissue. **Figure** **7**A shows the obtained HSOOL with a diameter of 3 mm and a height of 0.1 mm. The internal microstructure of the HSOOL exhibited uniform interconnected pores, and the pore size was ≈100 µm (Figure 7B; Figure S25, Supporting Information). The HSOOL exhibited a low swelling ratio in the range of 30–60% (Figure 7C). After 6 days, the cumulative amount of OPG released from HSOOL was 1.1 ng, and the total protein concentration in the released solution was 250 µg mL^−1^ (Figure 7D; Figure S26, Supporting Information). Compared to CLOO‐MCH, HSOOL had a relatively low release rate and degradation rate. More than 90% of CLOO‐MCH was degraded in 30 days, while HSOOL degradation took nearly 65 days (Figure S27, Supporting Information). However, HSOOL still achieved effective doses for regulating osteoclast/osteoblast balance in a short time (Figure S26, Supporting Information). To a certain extent, protein phosphorylation/dephosphorylation reflects protein activity; therefore, we examined the phosphorylation level of crosslinked proteins released from HSOOL. As shown in Figure S28, Supporting Information, we found no obvious differences in the phosphorylation levels of P‐ERK1/2 and P‐P38 before crosslinking (proteins in CLOO) and after crosslinking (proteins in HSOOL release solution), suggesting that the crosslinked proteins in HSOOL retained a degree of activity. In Figure S29A,C, Supporting Information, the rheological frequency sweep results presented show that the *G*′ values were higher than the *G*″ values in the range of 0.1–10 Hz, indicating that both CLOO‐MCH and HSOOL remained in a stable gel state in the measured frequency range. To compare the anti‐shear deformation ability of the hydrogel, a strain amplitude test was conducted to detect the critical point at which the collapse of the hydrogel occurred. As shown in Figure S27B,D, Supporting Information, HSOOL collapsed at a higher strain (260%) than CLOO‐MCH (20%). In addition. HSOOL was subcutaneously transplanted into the backs of mice to determine its biosafety. H&E staining images showed no obvious migration of macrophages or lymphocytes into the connective tissue around the HSOOL at 5 weeks (Figure S30, Supporting Information), indicating that HSOOL possessed excellent biocompatibility.

Furthermore, HSOOL, a hydrogel scaffold loaded with untreated osteocyte lysates (HSUOL), a blank hydrogel scaffold (Blank‐HS), anti‐ERK1/2 HSOOL (U126), and anti‐Wnt/*β*‐catenin HSOOL (DKK1) were transplanted into 10‐week‐old male mice that served as rodent calvarial defect models to investigate bone defect repair in vivo. As shown in Figure 7E, micro‐CT images indicated that the HSOOL significantly promoted bone healing compared to other treatment. clear homogeneous new bone formation occurred throughout the cranial defect and was integrated with the adjacent native bone. Moreover, quantitative analysis of the micro‐CT images revealed up to 77% bone healing in the HSOOL group (Figure 7F). In contrast, no obvious new bone formation was observed in the HSUOL (14.2%), Blank‐HS (7.5%), and Defect only groups (5.08%). Only partial bone healing was detected in the U126 (34.78%) and DKK1 groups (42.62%) (Figure 7E,F). BV/TV has been widely used to evaluate the quality of new bone formation. A significant increase in bone volume was detected in the HSOOL group (58.62%) compared with the defect‐only group and Blank‐HS group (3.636% and 5.732%, respectively) (Figure 7G). As shown in Figure 7H,I, and in Figure S31, Supplementary Information, H&E and Masson trichrome staining images revealed that HSOOL induced a larger newly mineralized region than the other treatments. In the defects treated with HSOOL, the surrounding calcified tissue presented striations, a characteristic of lamellar bone. However, in the corresponding Blank‐HS group, Defect only group and HSUOL group, the defects were filled with fibrous tissue or stromal‐like tissue with minimal bone formation. Compared to the HSOOL group, complete ossification was not obvious in defects treated with anti‐ERK1/2 HSOOL or anti‐Wnt/*β*‐catenin HSOOL. In the defects filled with HSOOL (Figure 7H), a large amount of new bone collagen (blue area, detected by Masson trichrome staining) was present both in the mid‐section of the defect site and in direct connection with the defect edge. However, no apparent new bone collagen was observed in the defect location in the Defect only group and the Blank‐HS group. In addition, compared to the HSOOL group, a significant decrease in the area of the new bone collagen formation region was detected in the U126 group and DKK1 group (Figure 7I). Collectively, these results demonstrated that HSOOL supported osteogenic differentiation and induced robust bone repair.

## 3 Conclusion

While osteocytes present promise for application in bone repair, osteocyte‐based therapy has not been used in clinical practice due to the difficulty of obtaining osteocytes with continuous and immobilized function. To overcome this problem, we developed a simple but highly effective approach to activate osteocytes with enhanced osteogenic induction ability on a large scale through OFF loading stimulation. In vitro studies demonstrated that the osteocyte lysates had superior advantages in immobilizing and preserving osteocytic regulatory function. We found that activated osteocyte lysates could induce a robust osteogenic response of osteoblasts and inhibit osteoclast activity in unloading and diseased environments. In addition, mechanistic studies revealed the key role of glycolysis and the ERK1/2 and Wnt/*β*‐catenin pathways in osteocytes during OFF loading induced bone remodeling. Moreover, the application of our designed osteocyte lysate‐based hydrogel was similar to creating an in situ stockpile of “activated osteocytes” to enrich and release functional molecules, thus persistently regulating endogenous osteoblast/osteoclast homeostasis and restoring bone injury. Notably, osteocytic activation, functional hydrogel design and the performance of osteocytic regulatory functions did not involve the use of defined media, inductive biochemicals, or highly specialized equipment. In summary, our findings provided a potential strategy to realize the translational use of osteocyte mechanotransduction‐mediated clinical bone regeneration and bone injury treatment.

## 4 Experimental Section

### MLO‐Y4 Cell Culture and Mechanical Stimulation

A total of 4 × 10^6^ MLO‐Y4 cells mL^−1^ were cultured in T75 cell culture flasks with Gibco *α*‐modified essential medium supplemented with 10% fetal bovine serum and 2% antibiotics (100 U mL^−1^ penicillin, 100 mg. mL^−1^ streptomycin) for 24 h under static culture conditions. To achieve osteocyte activation, many influencing factors were controlled, such as the initial cell density (3–4 × 10^6^ MLO‐Y4 cells mL^−1^), stressed area (75 cm^2^), fluid flow volume (8–10 mL), fluid velocity (0.15–0.6 m s^−1^), rocker plate amplitude (50 mm), revolutions per minute of the oscillating plate (55–125 rpm), and fluid flow stimulation time (4–48 h). Once the osteocytes (3rd–6th generation culture) reached ≈80% confluence in the T75 culture flask, the growth medium was refreshed, and the T75 culture flask was transferred into a CO_2_ oscillating incubator set at 37 °C with a 5% CO_2_ environment and 55–125 rpm oscillator frequency. The fluid flow rate was detected with a velocimeter. After stimulation for 4–48 h, the cells were collected, diluted in deionized water, and stored at −80 °C for further use.

### Preparation and Characterization of the Osteocyte Lysate‐Based Hydrogel

To prepare the injectable osteocyte lysate‐based hydrogel (CLOO‐MCH), whole‐cell lysates were prepared using OFF‐treated MLO‐Y4 cells by simply exposing the cells to deionized water followed by four consecutive freeze‒thaw cycles (frozen with liquid nitrogen and thawed in a 37 °C water bath). Then, the sediments from the lysed cells were centrifuged (50 × *g* for 13 min at 4 °C), and the supernatant was passed through a 0.22 µm filter to yield the cell extract, which subjected to a bicinchoninic acid (BCA) assay to determine the protein concentration. The final sample was stored at −80 °C until further use. Next, 10 wt% methylcellulose solution was first sterilized by UV light exposure for 0.5 h and then diluted in phosphate‐buffered saline (PBS) to prepare a 10 wt% methylcellulose solution. Then, the solution was passed through a syringe filter with a 0.22 µm pore size to further eliminate bacteria. Next, 25 µL of cell lysates (containing 0.6 mg protein) was homogeneously blended with 25 µL of 10 wt% methylcellulose solution in an ice‐water bath. Thermal crosslinking was induced by heating the composite hydrogel solution at 37 °C for 5 min. All experimental processes were performed under sterile conditions. The osteocyte lysate‐based hydrogel scaffold (HSOOL) was fabricated according to a previous study with some modifications.^[^ ^23^, ^24^ ^]^ Sodium alginate was dissolved in PBS at a concentration of 2 wt%. Briefly, 15 mg of EDC and 9 mg of NHS were added to 240 µL of alginate solution (2 wt%) and mixed well to activate the carboxylic acid group of sodium alginate. After 20 min, 20 µL of activated alginate solution was mixed with 30 µL of cell extract (containing 0.6 mg of protein) and rapidly injected into a cylindrical Teflon mold (*r* = 3 mm). After a complete gelling period of 24 h at −20 °C, the flaky porous scaffold was transferred to heating conditions to melt the ice crystals and then washed with PBS for 20 min at 4 °C to remove unreacted EDC and NHS. The cross‐sectional morphologies and macroporous structures of CLOO‐MCH and HSOOL were observed via scanning electron microscopy (SUPRA 60, Wavetest) and confocal microscopy. The mechanical properties were evaluated with an electronic universal material testing machine (MCR302, Anton Paar). The HSOOL was placed in PBS at 25 °C for measurement of the swelling rate. After swelling, excess liquid was removed from the hydrogels, and their weights were recorded at different time intervals (0.5, 1, 2, 3, 5, 25, 50, and 75 h). The equilibrium mass swelling rate (*S*_eq_%) was calculated using the following equation:^[^ ^38^ ^]^

(1) $$S_{\mathrm{eq}}\;\%=\frac{W_{\mathrm{eq}}-W_{d}}{W_{d}}\times 100\%$$

where *W*_eq_ is the mass of the swollen gel, and *W*_d_ is the mass of the dry gel.

The in vitro total protein and cytokines release profiles of CLOO‐MCH and HSOOL were determined in PBS at 37 °C at a 100 rpm shaking speed. The HSOOL (Φ 3 mm × 0.1 mm) and 50 µL of CLOO‐MCH solution were placed into a 1.5 mL tube with 1 mL of PBS. At predetermined time points, including 7, 12, 24, 48, 72, 96, 120, and 144 h, the PBS was collected and replaced with the same volume of fresh PBS. The total protein content released from the gels was measured with a BCA protein assay, and the OPG content released from the samples was determined using a mouse OPG enzyme immunoassay kit according to the manufacturer's protocol.

### CLOO‐MCH Stimulation of Osteoblasts and Osteoclasts In Vitro

Briefly, 50 µL of CLOO‐MCH solution was spread on the upper chamber of a Transwell (8 µm pore size), equilibrated at 37 °C for 15 min to form a soft gel film, and then immersed into cell culture medium at 37 °C for further use. Next, 3 × 10^4^ MC3T3‐E1 cells were seeded in each bottom chamber (24‐well plate) and cultured overnight, followed by replacement of the culture medium with fresh MM cm and cultivation with CLOO‐MCH within a fixed time period for further research. The medium was resupplied every 2 days. For CLOO‐MCH stimulation of osteoclasts, 2 × 10^3^ RAW 264.7 cells were cultured in each bottom chamber (24‐well plate) overnight, and then 40 ng mL^−1^ recombinant murine RANKL was added to the culture medium to induce osteoclast formation. After 2 days, CLOO‐MCH was immersed in the induction culture medium of osteoclasts in the upper chamber of the Transwell (8 µm pore size) to construct a culture system of RAW 264.7 cells with CLOO‐MCH.

### RNA Isolation, Quantitative Real‐Time PCR (qRT‐PCR) and mRNA‐seq

At the specified time points, total RNA was extracted from different cells (MC3T3‐E1, MLO‐Y4, RAW 264.7) using TRIzol reagent and then reverse‐transcribed to complementary DNA using a PrimeScript RT Reagent Kit with gDNA Eraser according to the manufacturer's protocol. qRT‐PCR was subsequently performed using the SYBR Green system in a Light Cycler 480 PCR instrument (Roche, IN, USA). *β*‐Actin was adopted as an internal control to normalize the signals of each target gene. The 2^−△△CT^ method was used for interpretation of the results. The primer sequences were detailed in Table S2, Supporting Information. To examine the mRNA expression profile of osteocytes, high‐throughput mRNA‐seq was performed on Illumina sequencing platforms. The RNA sequence data were imported into integrated differential expression and pathway analysis software (DESeq2 v1.26.0), and the target gene expressing the intensity more than onefold in oscillatory flow condition compared to those in the control group were indicated as normalized values by a color bar.

### Western Blotting Analysis

After the total protein content in cells was determined using a Bradford assay, the protein samples were separated in a 10% SDS‐PAGE gel and transferred onto a polyvinylidene fluoride membrane (Millipore, MA, USA). The membrane was blocked at room temperature in 5% skim milk for 1 h. Then, the membranes were incubated with primary antibodies overnight at 4 °C. The antibodies used in the present study were as follows: anti‐OPG, anti‐COX2, anti‐ERK1/2, anti‐P‐ERK1/2, anti‐Wnt3a, anti‐JNK, anti‐P‐JNK, anti‐p38, anti‐P‐P38, anti‐Runx2, anti‐OPN, anti‐IL‐6, anti‐TRAP, anti‐NFATc1, and anti‐OPG. The membranes were then washed with PBST and incubated with HRP‐conjugated secondary antibodies for 1 h at room temperature. After the membranes were washed with PBST three times, the immunoreactive bands were visualized with ECL detection reagent. The density levels of the detected proteins were normalized to the HSP‐90 or Actin expression level.

### Osteogenic Evaluation

To investigate the extent of osteogenic differentiation, ALP staining and Alizarin red staining were carried out. MC3T3‐E1 cells were fixed with 4% formaldehyde after coculturing with osteocyte lysates or osteocyte lysate‐based hydrogels for 7 days and then stained with BCIP/NBT working solution in the dark for 45 min, followed by washing with deionized water and imaging via optical microscopy. To quantify ALP activity, treated MC3T3‐E1 cells were digested in NP‐40 buffer and incubated in ALP buffer containing p‐nitrophenol phosphate substrate, and the absorbance was measured at 405 nm. To detect mineral deposition, an Alizarin red staining assay kit was used. After incubation with CLOO or CLOO‐MCH for 14 days, the MC3T3‐E1 cells were rinsed and stained with 2% Alizarin red staining solution for 1 h at room temperature to dissolve and release calcium‐bound Alizarin red. Subsequently, the cells were washed with deionized water three times, and the stained cells were observed and imaged with a light microscope. To quantify cell mineralization, Alizarin red‐stained cells were dissolved in 10% v/v acetic acid, and the absorbance was measured at 405 nm.^[^ ^40^ ^]^

### Osteoclast Cell Culture, TRAP Staining, and Bone Resorption Assay

To investigate the osteoclast formation capability, RAW 264.7 cells were seeded in 48‐well plates (1 × 10^3^ per well) and cultured with DMEM containing 10% FBS in a humidified CO_2_ incubator at 37 °C. On day 2, the medium was replaced with *α*‐MEM supplemented with 40 ng mL^−1^ recombinant murine RANKL, and the cells were cultured with 300 µg mL^−1^ CLOO or CLOO‐MCH. The medium was changed every 2 days. On day 7, the cells were fixed and stained using a TRAP staining kit. Images were captured under a microscope. The area of TRAP‐positive cells was calculated. For the resorption assay, 1.5 × 10^3^ RAW 264.7 cells per well were cultured and stimulated with RANKL from 0–9 days in 48‐well plates. At day 9, the induced osteoclasts were gently detached and seeded on the bottom chamber of a Transwell plate (4 × 10^4^ osteoclasts per well), in which cortical bovine bone slices (Φ 6 mm × 0.5 mm) had previously been placed. Subsequently, the upper chamber plated with CLOO‐MCH or filled with CLOO solution was cultured with the bottom chamber for another 7 days. At the end of the experiment, the bone slices were cleaned by ultrasonication at 40 kHz for 5 min in 70% alcohol, and the surface cells were scraped off with a brush, followed by rinses with PBS. The bone slices were stained with 1% toluidine blue for 5 min at room temperature, and the resorption pits were observed using a microscope. Meanwhile, the area of bone resorption was calculated to assess osteoclast activity.

### Measurement of Intracellular Ca^2+^

Ca^2+^ in MLO‐Y4 was detected using the Ca^2+^‐sensitive fluorescent dye Fluo‐4/AM. When oscillatory fluid shear stress was applied, 5 µm Fluo‐4/AM agent was added to the growth medium, followed by further incubation at 37 °C for 50 min. Then, the cells were washed with DPBS and cultured at 37 °C for an additional 30 min to allow thorough de‐esterification of the AM ester. The samples were then observed via inverted fluorescence microscopy.

### Determination of the Lactate Level of MLO‐Y4 Cells

The supernatant of MLO‐Y4 cell culture medium was collected after oscillating cultivation for 24 h and centrifuged at 2000 rpm to remove dead cells and cell debris. The supernatant (1:100 dilution) was then incubated with the working solution of the lactate assay for 30 min at 37 °C in the dark. Finally, the lactate level was determined by measuring the absorbance at a wavelength of 450 nm.

### CLOO‐MCH Reduced Bone Injury Induced by MBD In Vivo

All animal experimental procedures were performed strictly according to protocols approved by the Institutional Animal Care and Use Committee, Sun Yat‐Sen University (SYSU‐IACUC‐MED‐2022‐B0045). BALB/c‐nude mice were obtained from the Laboratory Animal Center of Sun Yat‐Sen University (Guang Zhou, China). Six‐week‐old male mice were assigned to different treatment groups: blank methylcellulose hydrogel (MCH); cell lysates from untreated osteocytes methylcellulose hydrogel (CLUO‐MCH); cell lysates from OFF‐treated osteocytes methylcellulose hydrogel (CLOO‐MCH); stimulation of osteocytes with the U126 inhibitor (U126); and stimulation of osteocytes with the DKK1 inhibitor (DKK1). To construct a myeloma model, the mice were first subjected to inhalational anesthesia with isoflurane, and then 2 × 10^6^ 5TGM1‐luc cells (constructed by the authors’ research group) were suspended in 10 µL of PBS and aspirated into a 50 µL microsyringe that was coupled to a 27‐gauge needle. The needle was inserted through the cortex of the anterior tuberosity of the left tibias. As the bone cortex was traversed, the needle was inserted 3–5 mm down the diaphysis of the tibia, and the cells were gently injected into the marrow space. The right tibia was also injected with 5TGM1‐luc cells at the same concentration as described above. On day 2, the establishment of myeloma was confirmed using an IVIS Spectrum Imaging System (PerkinElmer, USA). Then, 10 µL of MCH solution loaded with osteocyte lysates extract (containing 0.6 mg protein) was injected into the right tibias of the mice. For the left tibias, 10 µL of normal saline was injected as a negative control. Then, the tumor growth and location at 1 day, 10 days, and 20 days after the operation were monitored using the IVIS Spectrum system. The florescence intensity of tumor cells was quantified using ImageJ software. After 3 weeks, all mice were sacrificed, and their two tibias were removed and stored in 70% ethyl alcohol (EtOH) for micro‐CT analysis. The exposure parameters of the micro‐CT machine (Inveon, Germany) were set at 80 kV and 60 W. Micro‐CT Ray V3.0 software (Scanco Medical) was used to generate a 3D reconstruction of the metaphyses to evaluate bone mass. The bone volume, trabecula volume, and trabecula number were analyzed based on 200 contiguous slices within 1 mm proximity to the tibia according to a published study.^[^ ^39^ ^]^

### HSOOL Promotes Calvarial Bone Healing In Vivo

Ten‐week‐old male BALB/c‐nude mice were purchased from the Laboratory Animal Center of Sun Yat‐Sen University (Guang Zhou, China). All surgeries were performed under sterile conditions. Thirty‐five mice were randomly divided into 7 groups: blank hydrogel scaffold (Blank‐HS), hydrogel scaffold loaded with untreated osteocyte lysates (HSUOL), hydrogel scaffold loaded with OFF‐treated osteocyte lysates (HSOOL), osteocytes treated with the U126 inhibitor (U126), osteocytes treated with the DKK1 inhibitor (DKK1), and a defect only group. The surgical procedure for creating mouse calvarial defects was conducted according to a previous report with some modifications.^[^ ^40^, ^41^ ^]^ Briefly, general anesthesia was administered to the mice by injecting 3% pentobarbital sodium (50 mg kg^−1^), followed by administering 5% lidocaine hydrochloride to promote local anesthesia. Then, 3 mm circular full‐thickness calvarial defects were made on the skull of each mouse by a trephine drill with continuous irrigation, avoiding the build‐up of heat caused by friction. The bone pieces were carefully removed, the defect was covered with HSOOL (Φ 3 mm × 0.1 mm, containing 0.6 mg protein), and the gels were fixed using Vetbond medical glue. The skin incision was sutured. The other scaffold materials were regarded as a control group. Postoperatively, all the mice were transported to a heating pad until they fully recovered from anesthesia and were then returned to routine housing. Buprenorphine (0.1 mg kg^−1^) was administered for up to 3 days, and trimethoprim‐sulfamethoxazole was added to the drinking water for 7 days to prevent infection. Eight weeks after transplantation, entire calvarial bone tissues were collected and fixed in 4% paraformaldehyde (PFA) at 4 °C for 24 h and rinsed with normal saline. The specimens were soaked in 70% EtOH before scanning with a high‐resolution micro‐CT machine (Inveon, Germany) at 80 kV, 500 µA, 0.5 mm filtration, and 14 µm resolution. Micro‐CT Ray V3.0 software (Scanco Medical) was used to reconstruct the 3D structure of tibia and parietal bone samples. A region of interest (ROI) that was 2.5 mm in diameter was selected to analyze the trabecular bone and cortical bone. The images were obtained from the raw CT scanning data. The relative bone growth surface area (new bone area coverage) was calculated by using ImageJ software.

### Statistical Analysis

At least three independent experiments were performed in this study, and the values are presented as the mean ± SEM. The sample sizes in each group were 5 and 6 for statistical analysis of the results of animal experiments on calvarial defects and osteolysis, respectively. The numbers of biological replicates and technical replicates in each in vitro experiment were ≥3. Statistical significance was analyzed using GraphPad Prism 6.01 (GraphPad Software Inc.) *p* < 0.05 (*) was considered the cutoff for significance, 0.05 < *p* < 0.01 (**) was considered to indicate moderate significance, and *p* < 0.01 (***) was considered to indicate high significance. Multiple comparison tests were performed using one‐way analysis of variance (ANOVA).

## Conflict of Interest

The authors declare no conflict of interest.

## Supporting information

Supporting Information

## Data Availability

The mRNA‐seq Illimina reads for all sample data generated in this study have been deposited into the Genome Sequence Archive at the National Genomics Data Center under accession code CRA007474.The mass spectrometry identification data used in this study are available from the authors upon reasonable request, and all the other relevant data are available within the article and its supplementary information files.
