# Supplementary material for: Oscillating Fluid Flow Activated Osteocyte Lysate‐Based Hydrogel for Regulating Osteoblast/Osteoclast Homeostasis to Enhance Bone Repair
Source: Adv Sci (Weinh). 2023 Apr 5;10(15):2204592. doi: 10.1002/advs.202204592 (PMC10214251; doi:10.1002/advs.202204592)
Supplement: Supplementary file 1 — Supporting Information [file ADVS-10-2204592-s001.pdf]

ADVANCED SCIENCE
Open Access

Supporting Information

for *Adv. Sci.*, DOI 10.1002/advs.202204592

Oscillating Fluid Flow Activated Osteocyte Lysate-Based Hydrogel for Regulating Osteoblast/Osteoclast Homeostasis to Enhance Bone Repair

Liyuan Zheng, Disheng Zhou, Feier Ju, Zixuan Liu, Chenzhi Yan, Zhaoxia Dong, Shuna Chen, Lizhi Deng, Szehoi Chan, Junjie Deng and Xingding Zhang**

Supporting Information

Oscillating Fluid Flow Activated Osteocyte Lysate-based Hydrogel for Regulating Osteoblast/Osteoclast Homeostasis to Enhance Bone Repair

Liyuan Zheng, Disheng Zhou, Feier Ju, Zixuan Liu, Chenzhi Yan, Zhaoxia Dong, Shuna Chen, Lizhi Deng, Szehoi Chan, Junjie Deng, and Xingding Zhang*.*

Supplementary Methods
Supplementary Figures
Supplementary Tables

Materials and Methods

Sodium alginate (PRONOVA SLM, 100 G/M ratio ≤ 1) was purchased from Novamatrix (Norway), and 1-ethyl-3-(3-dimethylaminopropyl) carbodiimide hydrochloride (EDC), N-hydroxysuccinimide (NHS) and methylcellulose were purchased from Sigma–Aldrich (USA). MLO-Y4 cells, RAW 264.7 cells, 5TGM1 cells and MC3T3-E1 cells were obtained from the cell bank of the Chinese Academy of Science. Recombinant mouse Dkk-1 protein and receptor activator of nuclear factor *κ*B ligand (RANKL) were purchased from R&D Systems (USA). The primary antibodies presented in this article, including anti-PERK, anti-ERK1+ERK2, anti-phospho JNK, anti-JNK1+JNK2+JNK3, anti-p38, anti-IL-6, anti-Wnt3a, anti-OPG, anti-TRAP, anti-NFATC1, anti-Runx2, anti-OPN, anti-COX2, anti-HSP90, and anti-actin, were purchased from Abcam (UK). The inhibitors SP600125, SB352580, and U0126-EtOH were obtained from Selleck (USA). A cell plasma membrane staining kit with 3,3′-dioctadecyloxacarbocyanine perchlorate (DIO), a BCIP/NBT ALP color development kit and an Alizarin red staining kit were purchased from Beyotime (China). The alkaline phosphatase assay was purchased from Abcam (UK). The lactate assay kit-WST was purchased from Dojindo (Japan). The annexin v-APC/PI apoptosis kit was obtained from Multi Sciences (China). A tartrate-resistant acid phosphatase staining kit was obtained from Beibo (China). A variety of molecular biology agents, including PrimeScript RT Master Mix and SYBR Green reagent, were obtained from Bio-Rad (USA). The Ca^{2+}-sensitive resin dye Fluo-4 AM was purchased from Abcam (UK), and a mouse OPG ELISA kit was obtained from Signalway Antibody (USA). 2-(4-Amidinophenyl)-6-indolecarbamidine dihydrochloride was purchased from Sigma (USA), and a tartrate-resistant acid phosphatase (TRAP) staining kit and Masson Goldner trichrome kit were supplied by Bestbio (China).

Effect of CLOO on osteoblast proliferation, morphology, migration and gene expression

First, the culture supernatant of murine myeloma cells (5TGM1) was collected and used as the conditioned culture medium (MM cm) to stimulate preosteoblast cells (MC3T3-E1). Then, CLOO was diluted with culture medium or a mixed solution of MM cm and normal culture medium (1:1) to a concentration of 300 μg mL^{-1}. Then, 2×10^3 MC3T3-E1 cells were seeded per well of a 96-well plate and cultured overnight. Subsequently, the culture medium was replaced with 200 μL of MM cm or CLOO, and after 24 h of stimulation, MC3T3-E1 proliferation was observed via a CCK-8 assay. To investigate whether CLOO can protect osteocytes from apoptosis in the MM microenvironment, 4×10^4 MC3T3-E1 cells were planted and cultured in 6-well plates (untreated surface) for 12 h and then cocultured with 2×10^5

5TGM1 cells under CLOO (total protein concentration = 300 μg mL^{-1}) stimulation for 5 days. After that, the suspension of 5TGM1 cells was removed, and MC3T3-E1 cells were collected for apoptosis analysis using a flow cytometer. The migration of MC3T3-E1 cells was evaluated by a Transwell assay. In brief, 400 μL of CLOO (total protein concentration = 300 μ*g* mL^{-1}) solution was added to the lower chambers, and 2×10^4 MC3T3-E1 cells were seeded in the upper chambers with 200 μL of medium containing 5% serum. After reaching the determined culturing time (24 or 48 h), the cells were fixed, stained and imaged by microscopy. The morphology of MC3T3-E1 cells during differentiation was visualized using an inverted fluorescence microscope after staining with DIO and DAPI after 24 h of CLOO stimulation.

Proteomic analysis of MLO-Y4 cells

LC–MS/MS was performed to analyze the differentially expressed proteins in MLO-Y4 cells with or without OFF loading. Briefly, the total cell lysates from the OFF loading group and untreated group were collected, and the protein content was quantified. Equal amounts of protein were loaded on a gel and separated by 12% SDS–PAGE, followed by Coomassie blue staining. The protein bands with a significant change in color intensity were excised from the gel and identified by LC–MS/MS technology (China). Subsequently, the cellular location and function of these differentially expressed proteins were classified.

Inhibitor Studies

A total of 4×10^6 MLO-Y4 cells were seeded in T75 cell culture flasks for 12 h, followed by treatment with culture medium containing different inhibitors (SP600125, SB352580, U126, DKK1) at working concentrations (Table S2). After being stimulated by the inhibitors for 1 h under static culture conditions, the cells were transferred into a CO_2 oscillating incubator for another 24 h of OFF loading culture. Finally, the OFF-treated cells were collected and processed into cell lysates for future use.

Bone histological analyses

The bone tissue (tibia and parietal bone) was fixed in 4% PFA for 24-48 h at 4 °C and demineralized with EDTA decalcifying solution (Boster, China) at 37 °C with gentle shaking for up to 2 weeks. Paraffin-embedded bone tissue was sliced into 3-μm-thick sections and subjected to H&E and TRAP activity staining assays, which were used to assess the trabecular bone and osteoclast distribution *in vivo*. Masson’s trichrome staining was used to evaluate

osteoblast and new bone formation *in vivo*. Images of tissue sections were captured by a microscope.

Rheological measurements

The temperature sweep oscillatory shear measurements were performed on an ARES/RFS II rheometer (TA instruments, USA) using a 10 mm parallel-plate geometry between 15 and 55 °C and with a temperature interval of 2 °C. A shear stress of 10 Pa and an oscillatory frequency of 1 Hz were applied. Storage (G′) and loss (G″) moduli were determined using a frequency sweep test (0.1-10 rad s^{-1} frequency). The critical point at which the collapse of the hydrogel occurred was determined via the strain amplitude sweep test (0.1-1000%) at 1 rad s^{-1}

Characterization of CLOO-MCH and HSOOL

To characterize the surface morphology and the microstructure of CLOO-MCH and HSOOL, the freeze-dried CLOO-MCH and HSOOL were sputter-coated with gold for 45 s and were then observed using a scanning electron microscope (SUPRA 60, Wavetest) at an acceleration voltage of 15 kV.

***In vivo* degradation and biocompatibility of HSOOL and CLOO-MCH**

The HSOOL (Φ 3 mm × 0.1 mm) scaffold and 50 μL of CLOO-MCH were implanted into the subcutaneous tissues of BALB/c nude mice (~ 20 g). The mice were humanely sacrificed at 10, 20, and 30 days. The implantation regions on the back skin of the mice were carefully opened, and the remaining gels were photographed. The skin tissue was surgically removed from the area surrounding the implant and subjected to H&E staining for tissue response analysis.

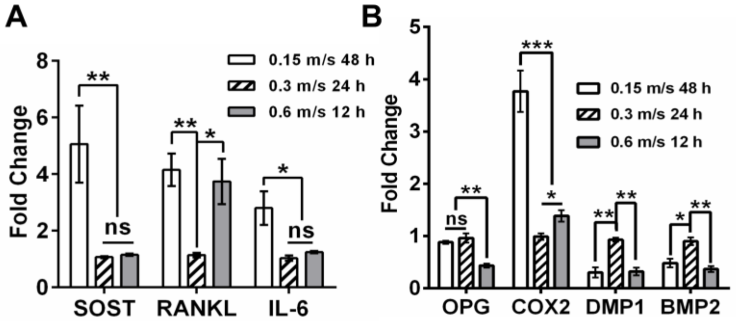

Supplementary Figure 1. **A**, The mRNA expression of pro-osteoclast factors and **B**, bone growth factors in osteocytes with OFF stimulation under different flow velocities with different stimulation times. Each error bar represents the mean ± SEM of three independent experiments. **p*<0.05, ***p*<0.01, ****p*<0.001; ns, not significant.

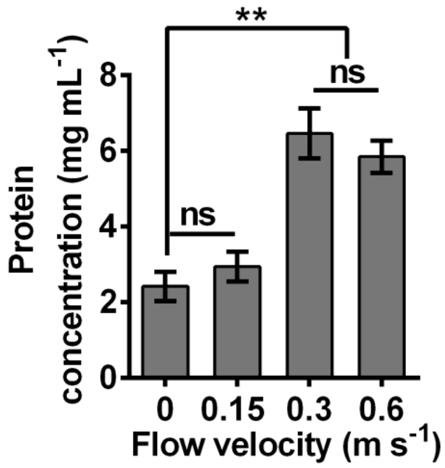

Supplementary Figure 2. The total protein concentration in osteocytes with OFF loading at different flow velocities. Each error bar represents the mean ± SEM of three independent experiments. *p<0.05, **p<0.01, ***p<0.001; ns, not significant.

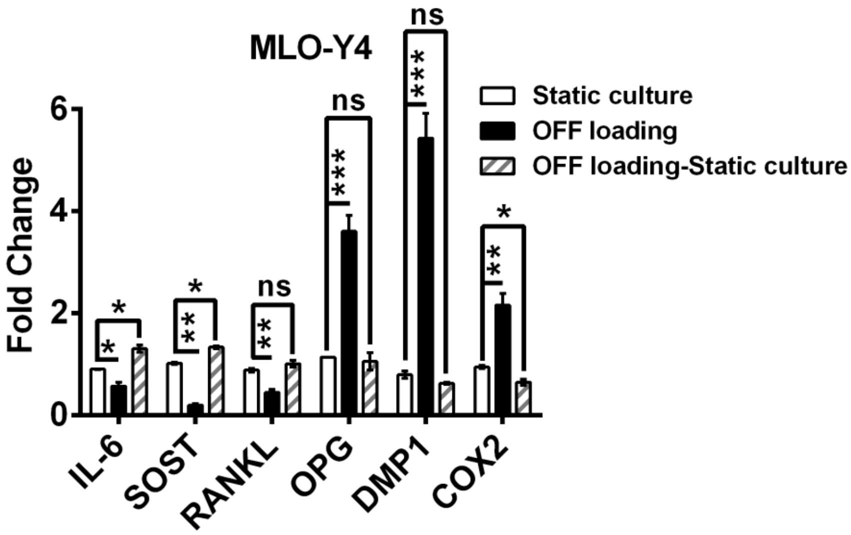

Supplementary Figure 3. Osteocyte-specific gene expression under different culture conditions. Each error bar represents the mean ± SEM of three independent experiments. **p*<0.05, ***p*<0.01, ****p*<0.001; ns, not significant.

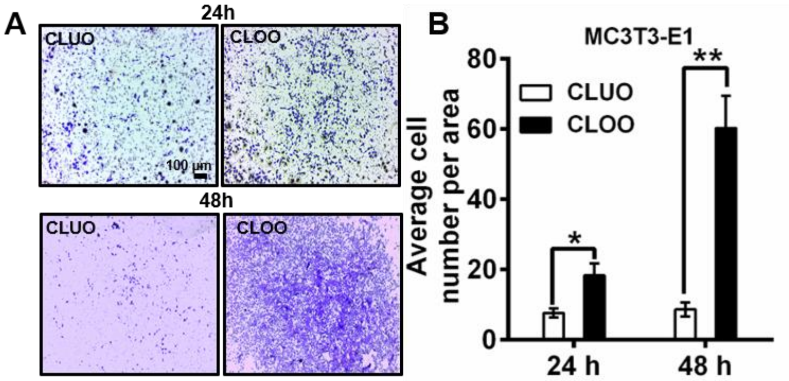

Supplementary Figure 4. Transwell invasion assays of migrating osteoblasts. **A**, The effect of CLOO and CLUO treatment on MC3T3-E1 cells migration at 24 h and 48 h. **B**, The number of migrating MC3T3-E1 cells per area at different time points. The data are expressed as the mean ± SEM (n=3 per group). **p* < 0.05, ***p* <0.01.

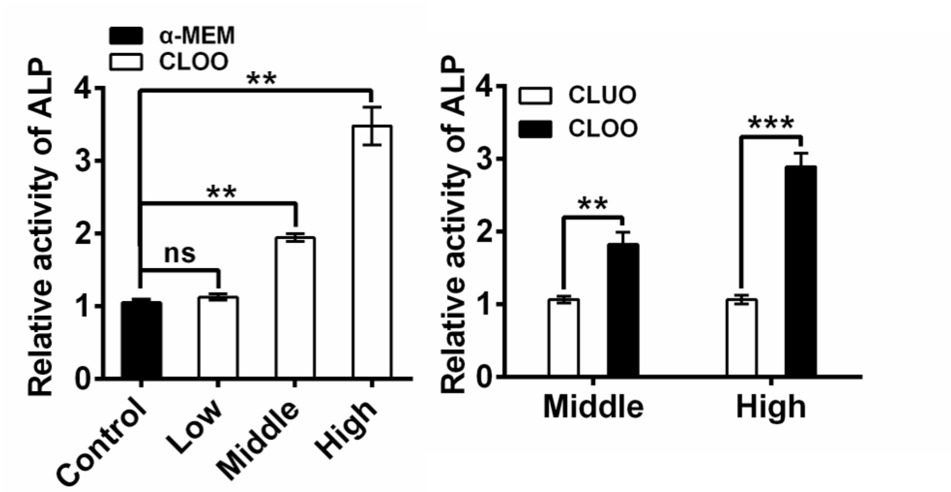

Supplementary Figure 5. ALP activity in MC3T3-E1 cells treated with CLOO and CLUO under different concentration. Each error bar represents the mean ± SEM of three independent experiments. *p < 0.05, **p <0.01, ***p<0.001; ns, not significant.

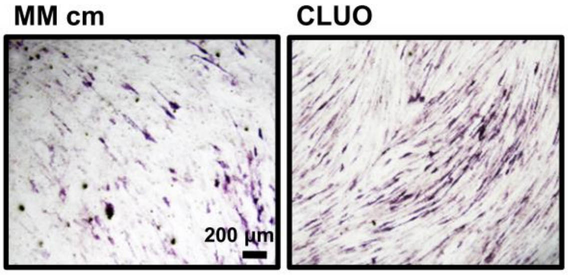

Supplementary Figure 6. ALP expression in MC3T3-E1 cells visualized by ALP staining at day 8. (n=4 images per group).

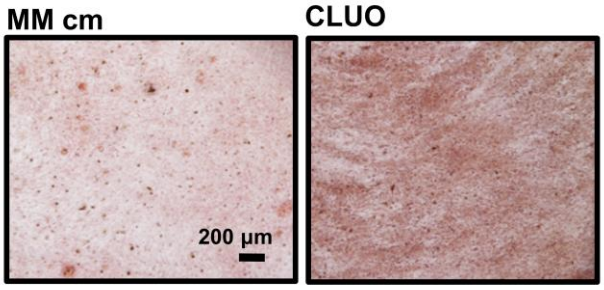

Supplementary Figure 7. Mineralization of MC3T3-E1 cells was assessed by Alizarin red staining at day 16. (n=4 images per group).

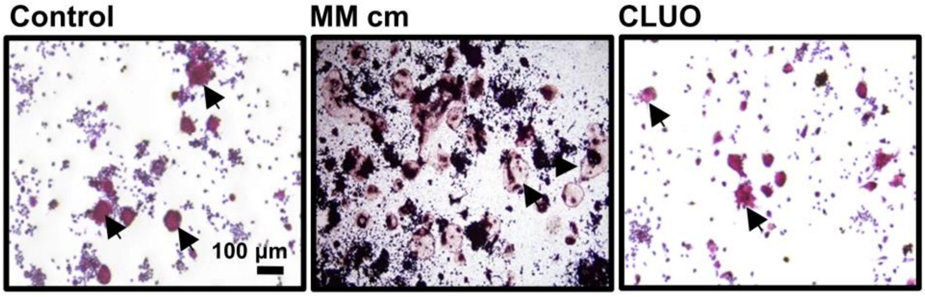

Supplementary Figure 8. Representative images of TRAP staining after 7 days of coculture. Black arrows represent positive multinucleated osteoclasts. (n=3 images per group).

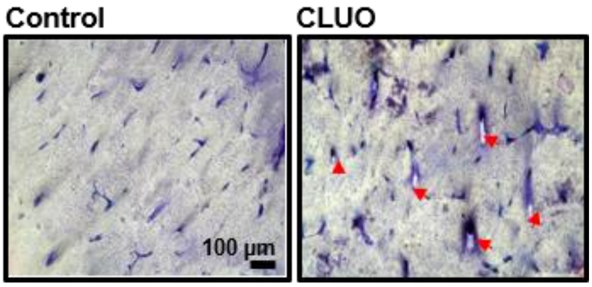

Supplementary Figure 9. Representative images of bone resorption after 7 days of coculture. Red arrows represent etching bone defects. (n=3 images per group).

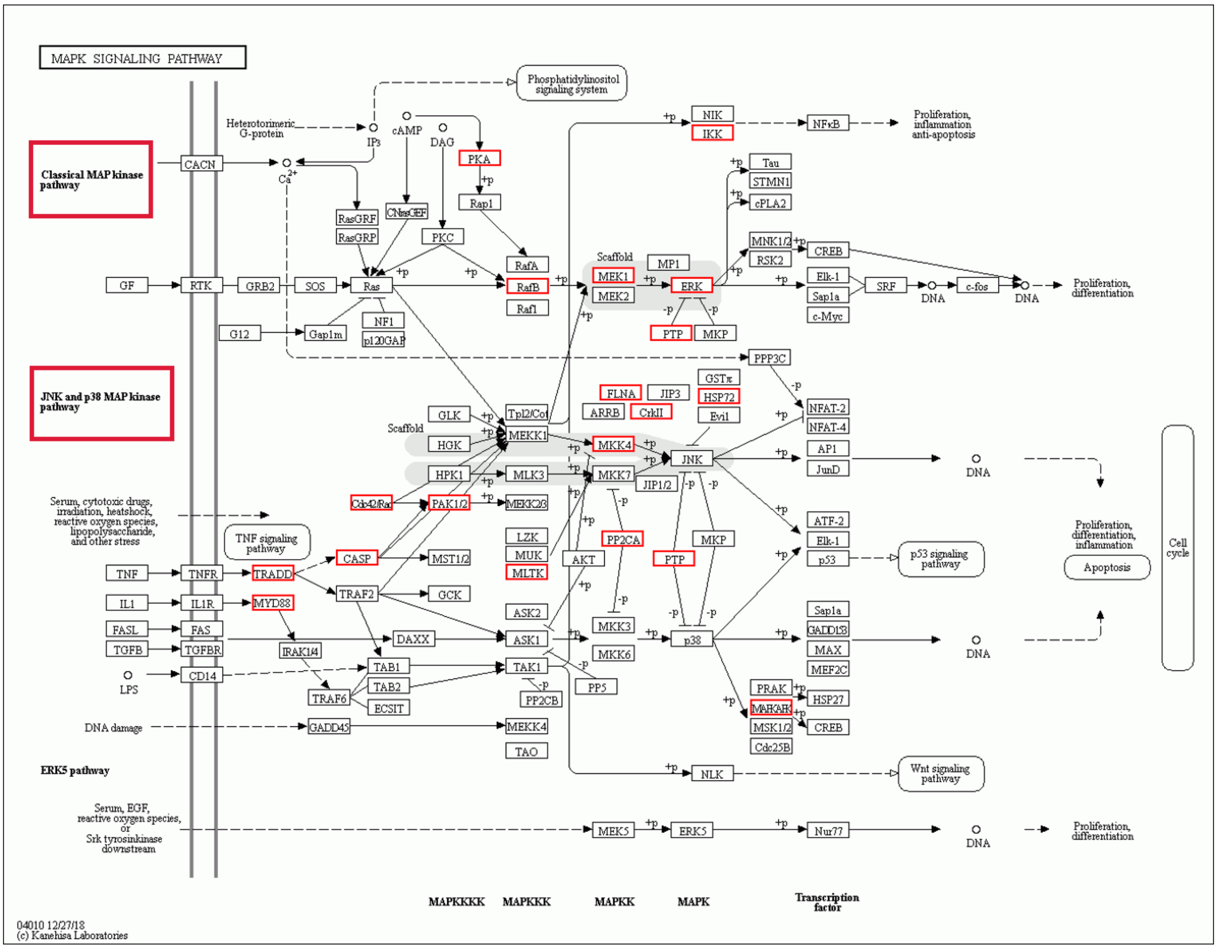

Supplementary Figure 10. Network diagram of the MAPK signaling pathway. The small red rectangle represents the uniquely identified proteins in MLO-Y4 cells subjected to OFF loading.

A

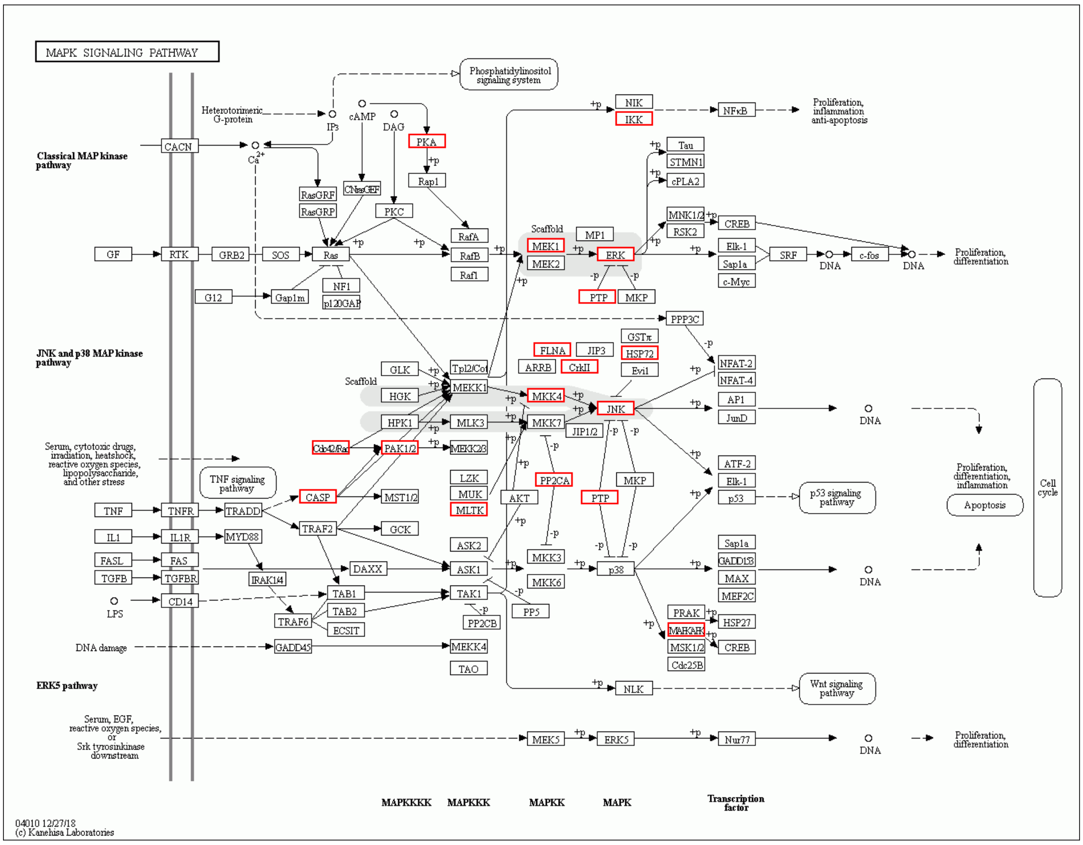

B

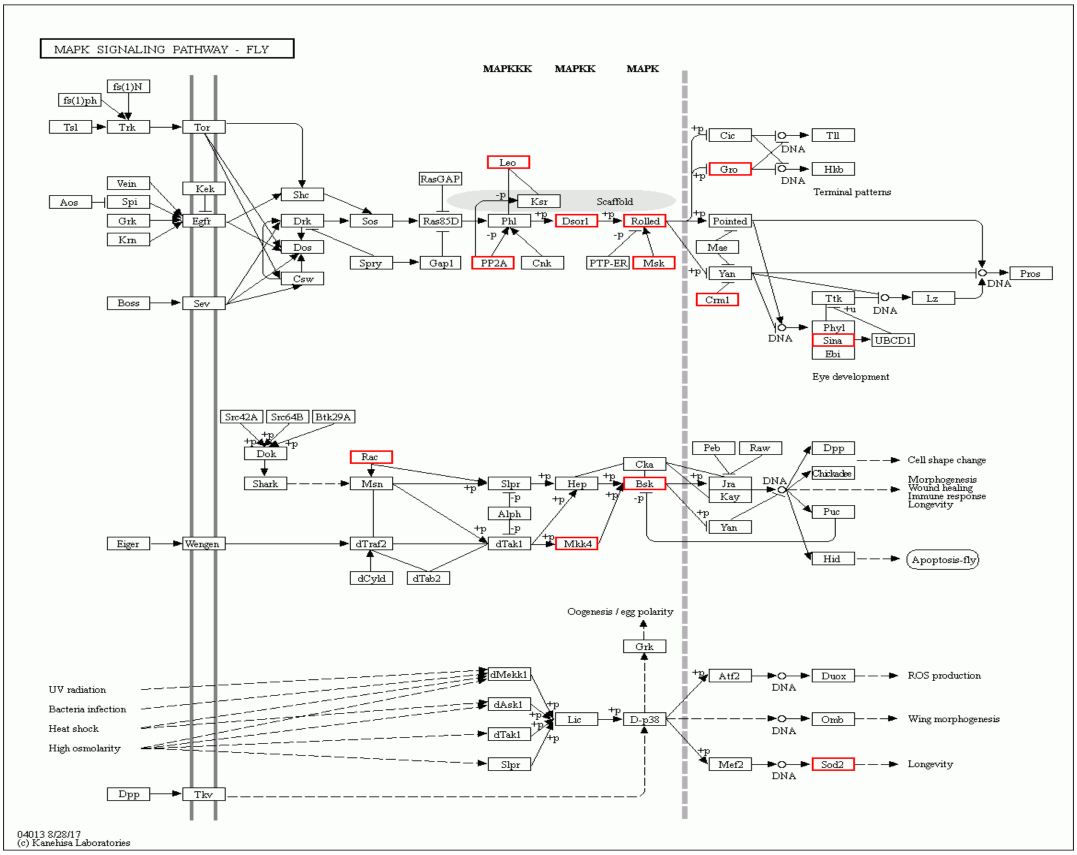

Supplementary Figure 11. **A,** Network diagram of the MAPK signaling pathway and **B,** MAPK-fly signaling pathway. The small red rectangle represents the uniquely identified proteins in MLO-Y4 cells subjected to OFF loading.

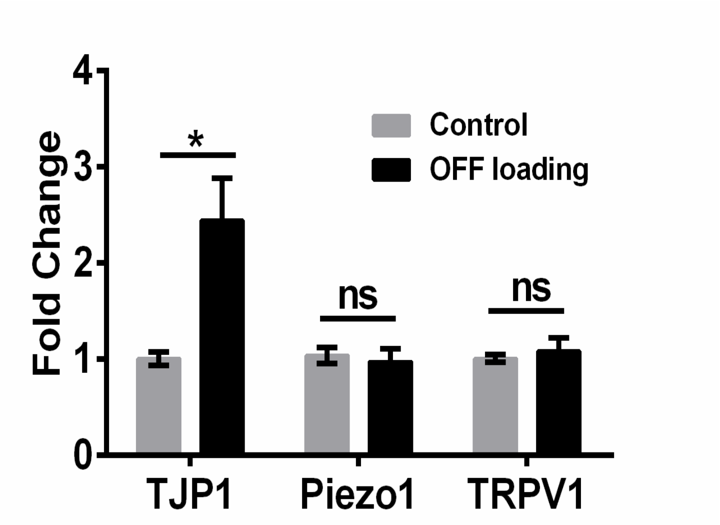

Supplementary Figure 12. The expression of TJP1, Piezo1 and TRPV1 in osteocyte was measured by real-time PCR under OFF loading condition or not. Each error bar represents the mean ± SEM of three independent experiments. *$p < 0.05$; ns, not significant.

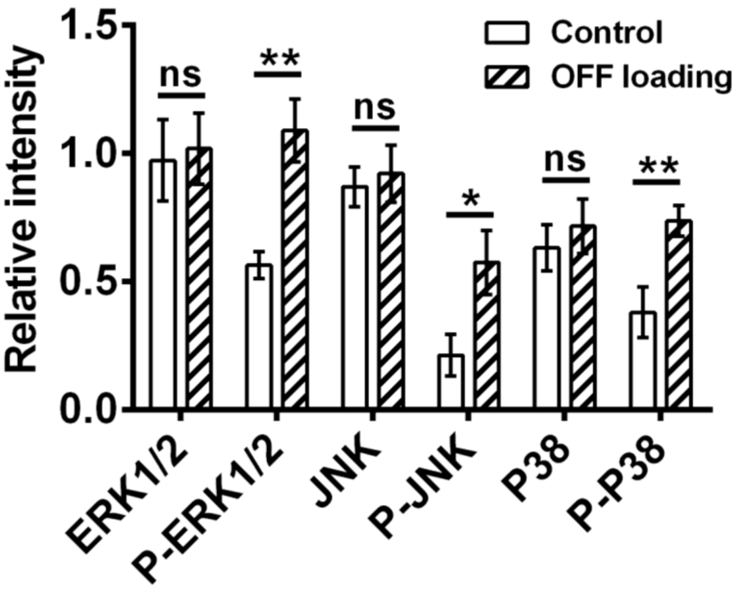

Supplementary Figure 13. Western blot analysis of ERK1/2, P-ERK1/2, JNK, P-JNK, P38 and P-P38 expression.

Control **OFF loading**

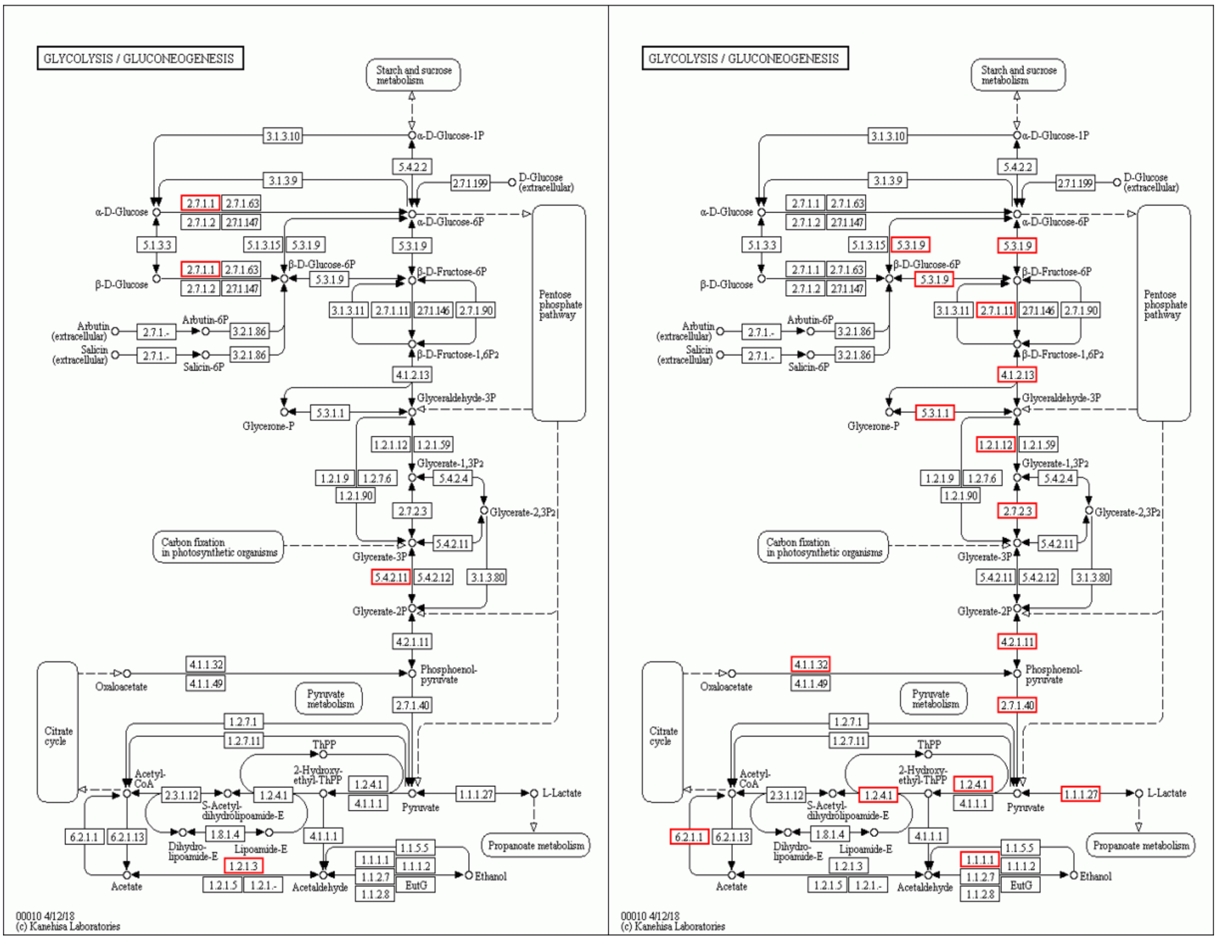

Supplementary Figure 14. Network diagram of the glycolysis/gluconeogenesis pathway. The small red rectangle represents the uniquely identified proteins in osteocytes (MLO-Y4) with or without OFF loading.

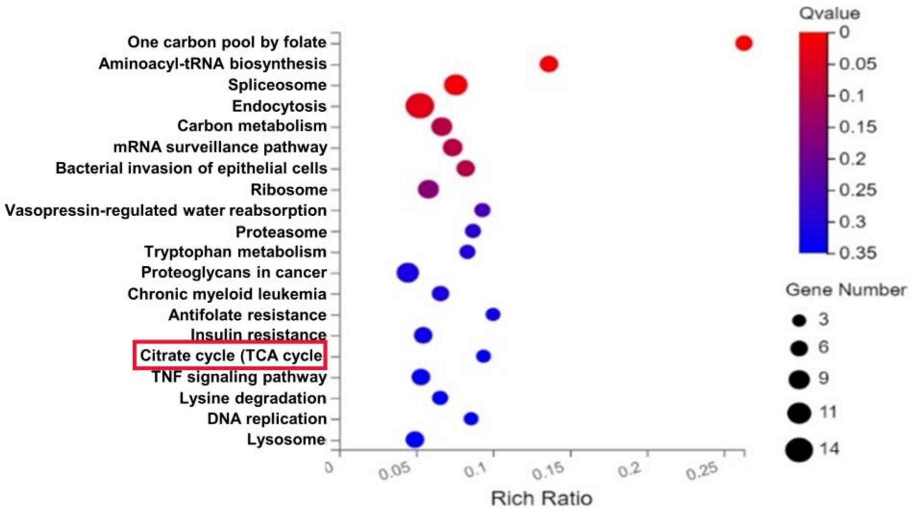

Supplementary Figure 15. KEGG pathway enrichment analysis and prediction of the function of specifically expressed proteins in osteocytes without OFF loading.

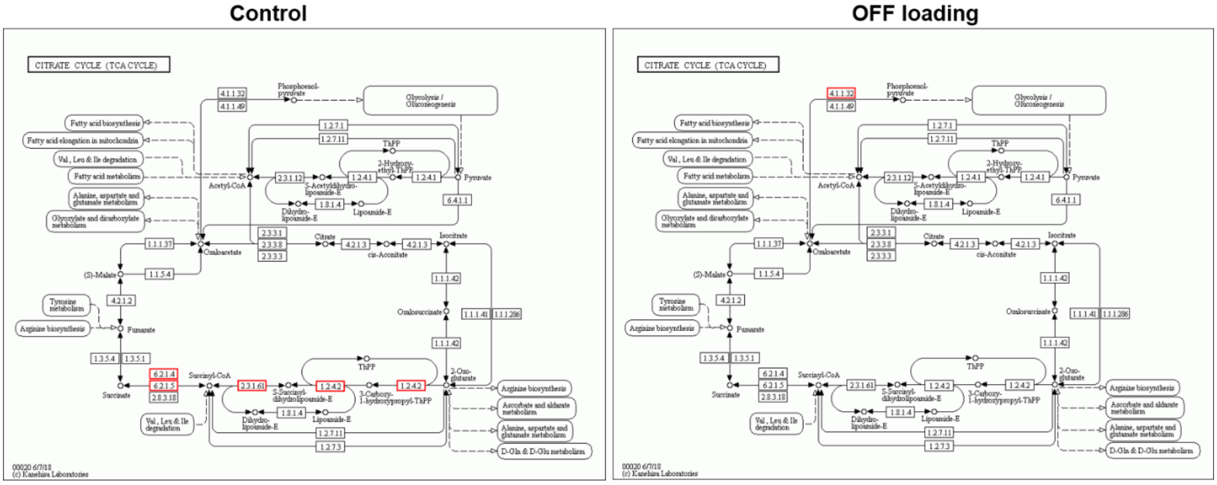

Supplementary Figure 16. Network diagram of the citrate cycle (TCA cycle) pathway. The small red rectangle represents the uniquely identified proteins in osteocytes (MLO-Y4) with or without OFF loading.

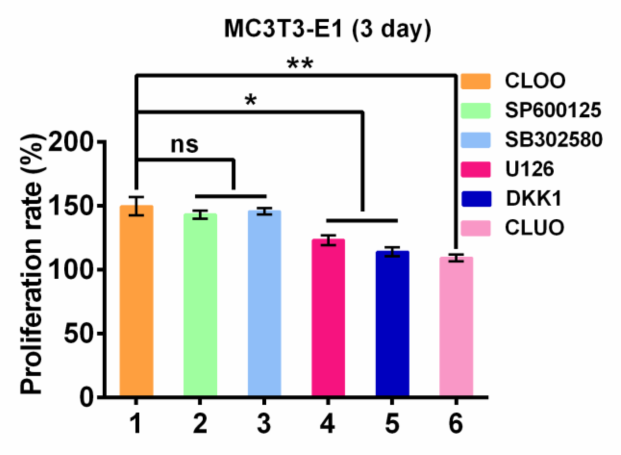

Supplementary Figure 17. Proliferation rate of MC3T3-E1 cells on day 3. Specific ERK1/2 and Wnt/*β*-catenin inhibitors (U126 and DKK1) blocked the increase in osteoblast growth induced by CLOO. Each error bar represents the mean ± SEM of three independent experiments *p < 0.05, **p <0.01, ***p<0.001; ns, not significant.

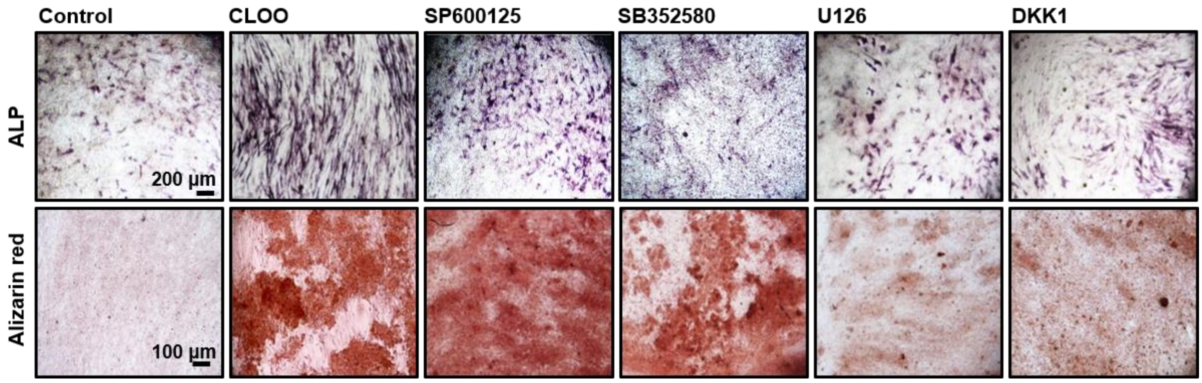

Supplementary Figure 18. The positive effect of CLOO on ALP expression and mineralization was reversed by inhibition of the ERK1/2 (U126) and Wnt/β-catenin (DKK1) signaling pathways. (n=3 images per group)

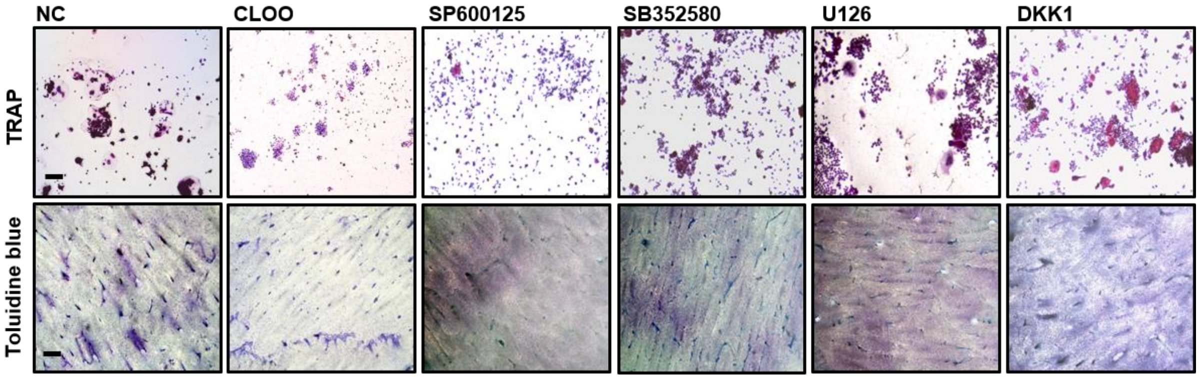

Supplementary Figure 19. Osteoclasts were stained for TRAP activity. Following osteoclasts removal, bone resorption pits were visualized by Toluidine blue staining. The negative effect of CLOO on osteoclast activity was reversed by pretreating osteocytes with U126 and DKK1. Scale bar: 200 μm. (n=3 images per group)

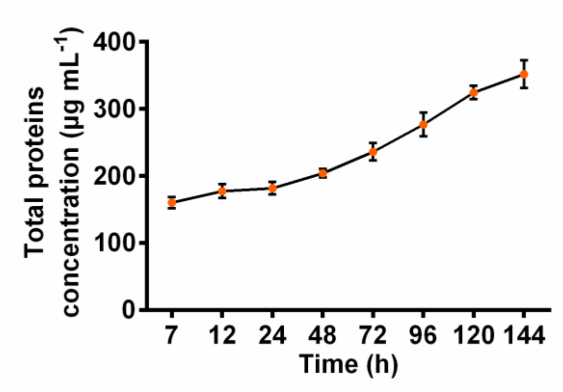

Supplementary Figure 20. Accumulative release profiles of total proteins from CLOO-MCH. (n=3 per group)

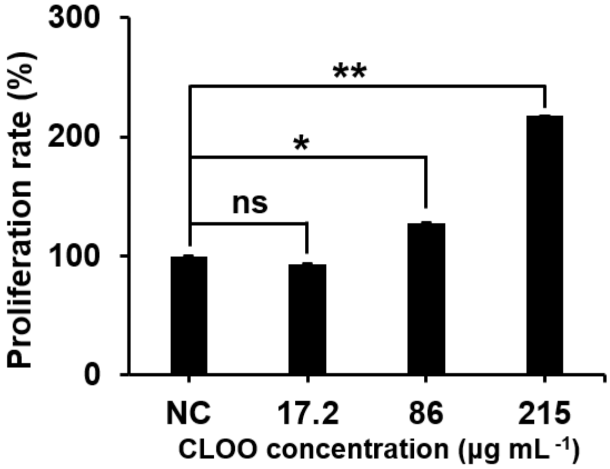

Supplementary Figure 21. CCK8 analysis of the viability of MC3T3-E1 cells treated with CLOO at day 1. Each bar represents the mean ± SEM of three independent experiments. $*p < 0.05$, $**p < 0.01$, $***p < 0.001$; ns, not significant.

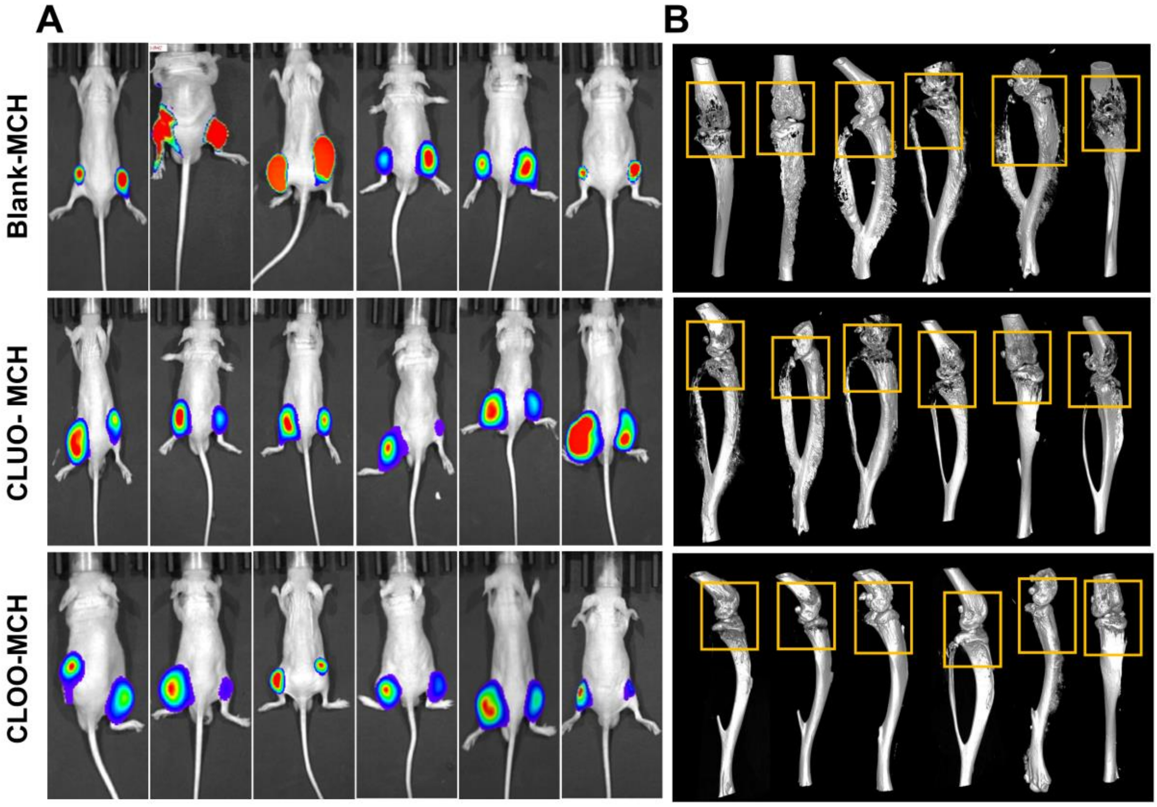

Supplementary Figure 22. **A**, Bioluminescence images of the tibia tissue at 20 days post-injection with 5TGM1-luc cells and different types of hydrogels. **B,** Representative 3D reconstructive images of the collected tibia from different groups.

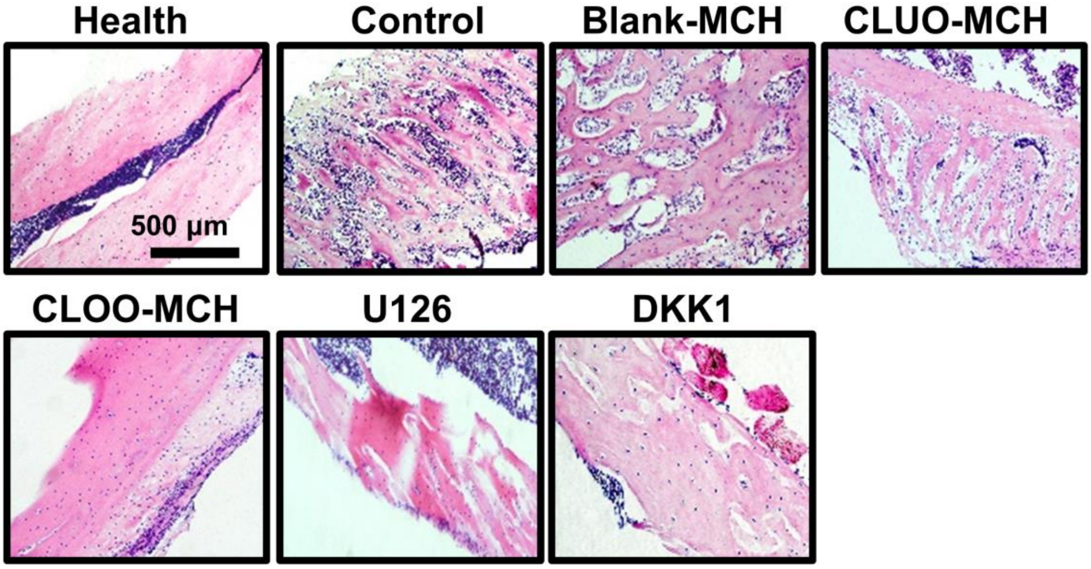

Supplementary Figure 23. H&E staining of tibia sections collected from mice in the indicated group after 20 days of post-injection with 5TGM1-luc cells and different types of hydrogels in mice.

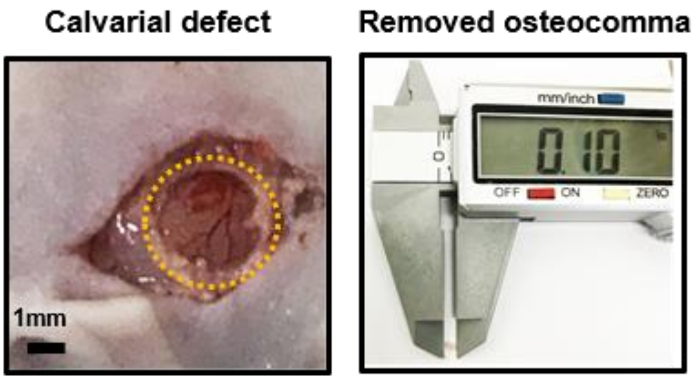

Supplementary Figure 24. Photograph of the exact surgical wound size made in BALB/c-nude mice.

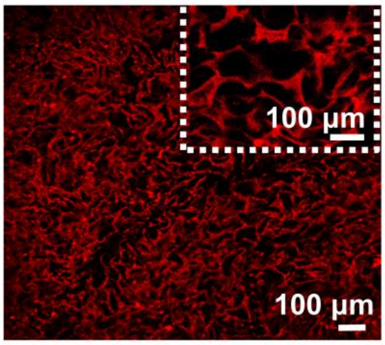

Supplementary Figure 25. A confocal image of HSOOL. The inset image within the white dotted box is a representative magnified image. HSOOL was labeled with Rho-BSA (red).

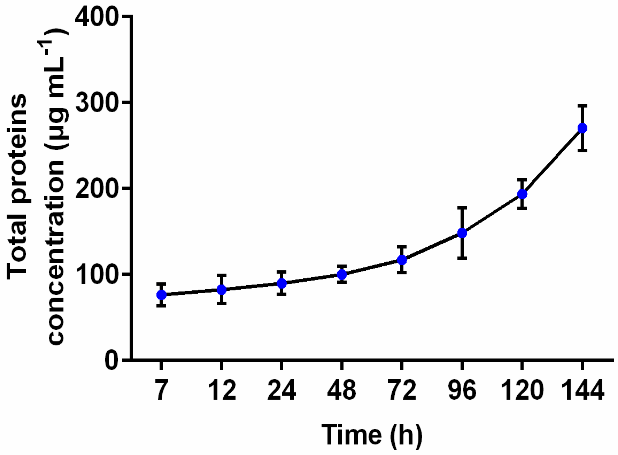

Supplementary Figure 26. Accumulative release profiles of total proteins from HSOOL (n=3 per group).

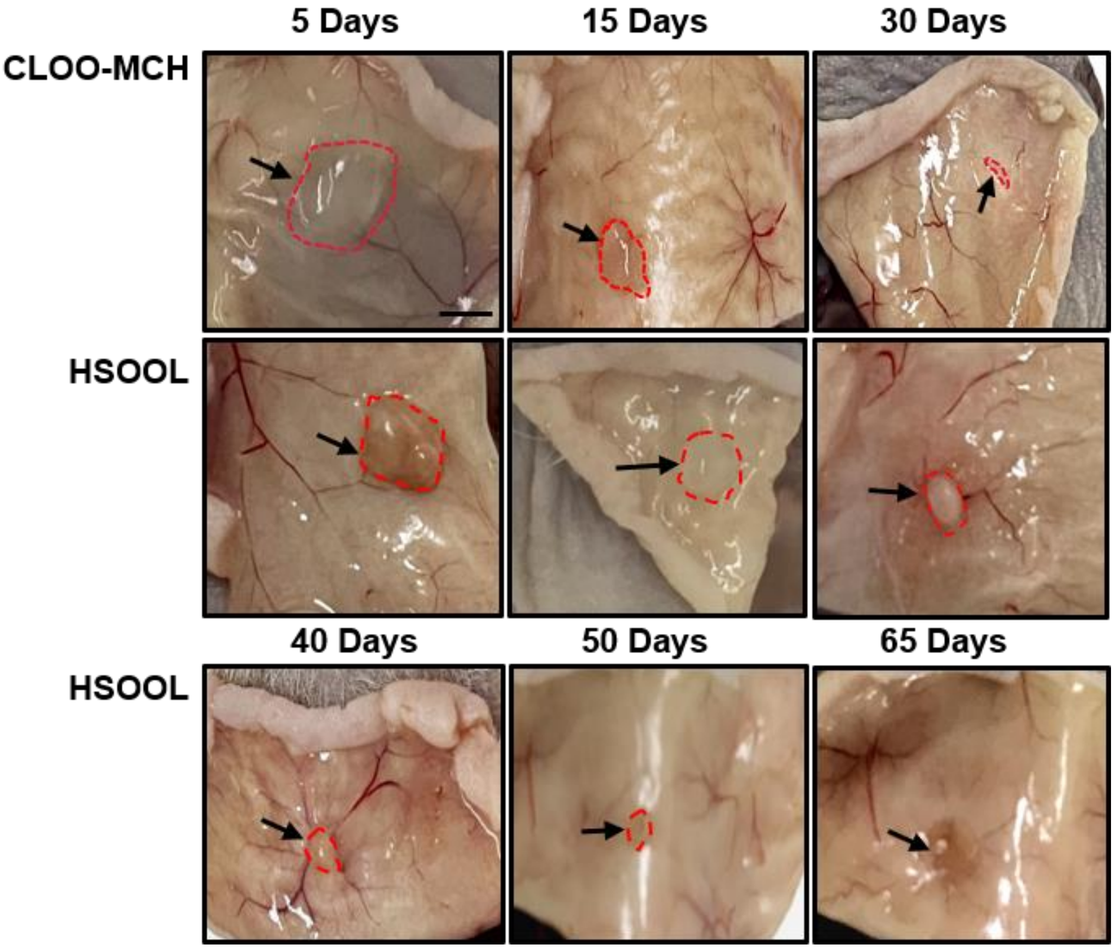

Supplementary Figure 27. Photographs of the *in vivo* degradation behavior of CLOO-MCH and HSOOL at different time points. Scale bar: 5 mm (n=3 animals per group).

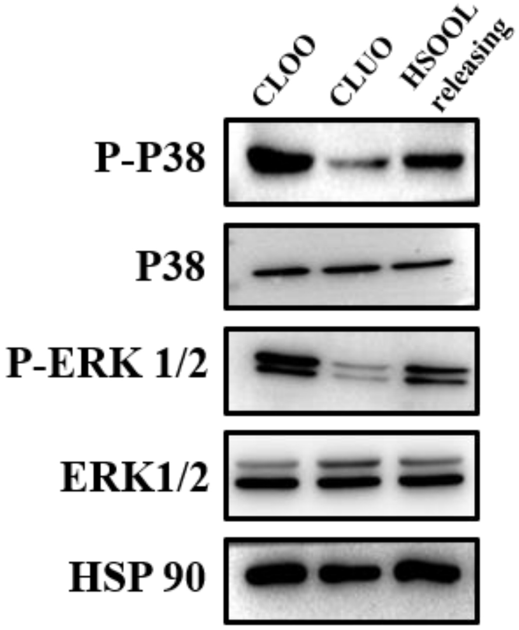

Supplementary Figure 28. Western blotting analysis of the protein phosphorylation level.

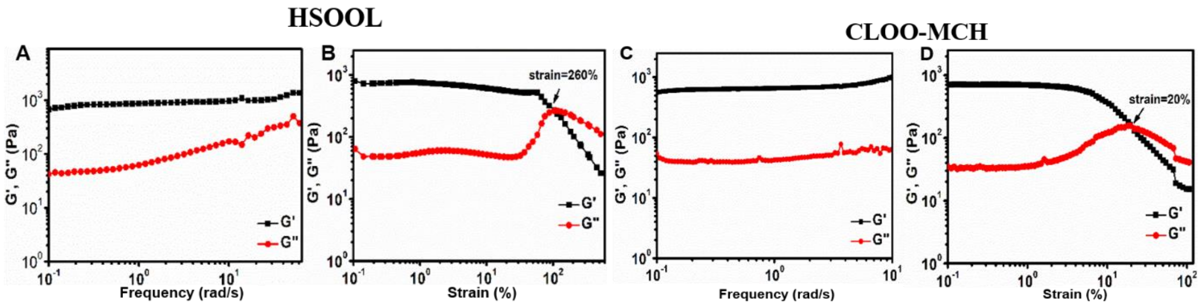

Supplementary Figure 29. **A**, **C,** Rheological data from the frequency sweep test. G′ and G′′ values of **B**, HSOOL and **D**, CLOO-MCH from the strain amplitude sweep test. n=3 hydrogels for each group.

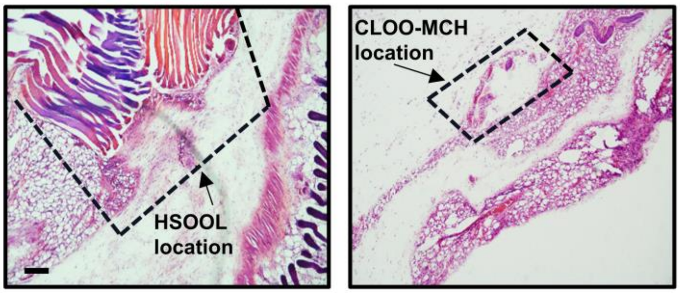

Supplementary Figure 30. H&E staining of the osteocyte-based hydrogels with tissues after 5 weeks of transplantation and post-injection. Scale bar: 500 μm (n=3 images per group).

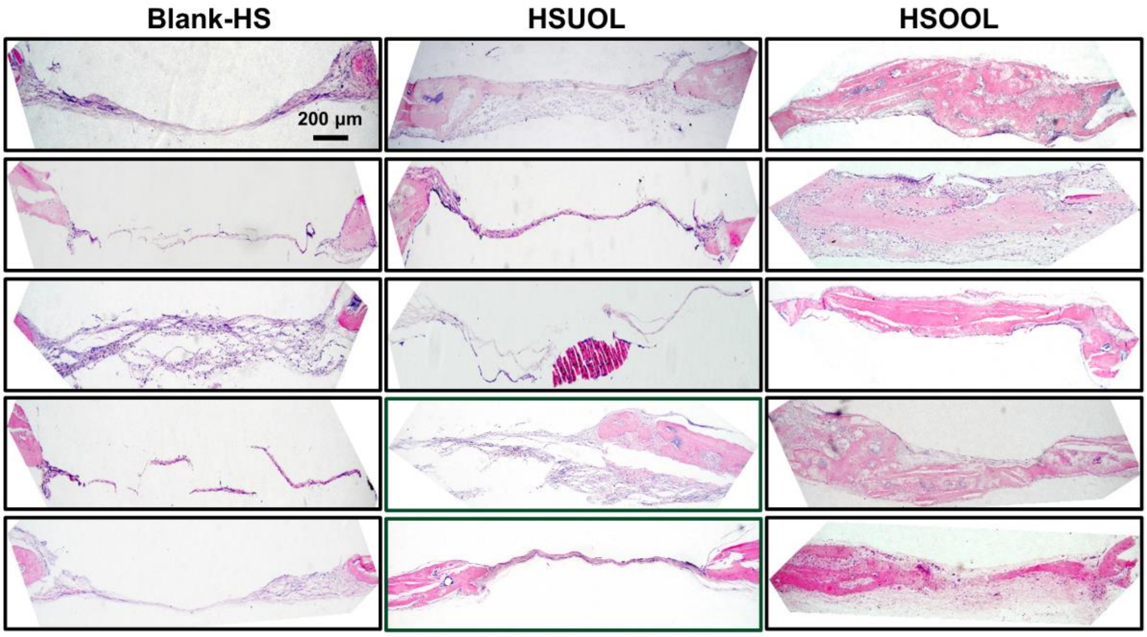

Supplementary Figure 31. Representative images of H&E staining of cranial bone in mice after 8 weeks of transplantation. (n=5 animals per group)

Supplementary Table 1. List of Inhibitors and Working Concentrations.

Function	Inhibitors	Working concentration
ERK inhibitor	U126	10 μM
P38 inhibitor	SB352580	10 μM
JNK inhibitor	SP600125	5 μM
Wnt/*β*-catenin inhibitor	DKK1	30 μg / mL

Supplementary Table 2. Sequences of primers used for qRT–PCR.

Gene name	Forward primer	Reverse primer
β-actin	GGCTGTATTCCCCTCCATCG	CCAGTTGGTAACAATGCCATGT
BSP	CAGGGAGGCAGTGACTCTTC	AGTGTGGAAAGTGTGGCCTT
OPN	AGCAAGAAACTCTTCCAAGCAA	GTGAGATTCGTCAGATTCATCCG
ALP	TGGCACGTTAAAGGTAATCAG	GAATCAAATGTTCAGGGTGGT
OPG	CTGCAATACACACACTCATCACT	ACCCAGAAACTGGTCATCAGC
OCN	GGGAGACAACAGGGAGGAAAC	CAGGCTTCCTGCAGGTACCT
COL 1	GCTCCTCTTAGGGGCCACT	CCACGTCTCACCATTGGGG
Runx2	CCAACCGAGTCATTTAAGGCT	GCTCACGTCGCTCATCTTG
SOST	AGCCTTCAGGAATGATGCCAC	CTTTGGCGTCATAGGGATGGT
NFATc1	CCGCTGGGAACAGCGATAG	CCGGACGCTGTCTCTCCAGGTGG
CTSK	TGGATGAAATCTCTCGGCGT	TCATGTCTCCCAAGTGGTTC
ACP5	CACTCCCACCCTGAGATTTGT	CATCGTCTGCACGGTTCTG
TRAP	TACCTGTGTGGACATGACC	CAGATCCATAGTGAAACCGC
MMP9	TCCAGTACCAAGACAAAGCCTA	TTGCACTGCACGGTTGAA
RANK	AGAGATGAACGTGGAGTTACTCTTT	CCAGTCAAGCAGCAGCCAGCAT
RANKL	TCCAGAAGACCAGAGGAAAT	GGAAGGGTTGGACACCTGAATG
IL-6	CCAAGAGGTGAGTGCTTCCC	CTGTTGTTCAGACTCTCTCCCT
COX2	TTCAACACACTCTATCACTGGC	AGAAGCGTTTGCGGTACTCAT
DMP1	CATTCTCCTTGTGTTCCTTTGGG	TGTGGTCACTATTTGCCTGTG
BMP2	GGGACCCGCTGTCTTCTAGT	TCAACTCAAATTCGCTGAGGAC
Wnt1	GGTTTCTACTACGTTGCTACTGG	GGAATCCGTCAACAGGTTCGT
Wnt3a	CTCCTCTCGGATACCTCTTAGTG	GCATGATCTCCACGTAGTTCCTG
Wnt5a	CAACTGGCAGGACTTTCTCAA	CATCTCCGATGCCGGAACT
β-catenin	ATGGAGCCGGACAGAAAAGC	CTTGCCACTCAGGGAAGGA
C-jun	GGATCAAGGCGGAGAGGAA	TGCAACTGCTGCGTTAGCAT
Cyclin-D1	GCGTACCCTGACACCAATCTC	CTCCTCTTCGCACTTCTGCTC
JNK	GAAGTGCAAACCTTGCGTCAGTA	AATAAGCGTATGCAGCCAATTCC
P-38	GATGAGCCTGTTGCTGACCCTTA	TGGTGGCACAAAGCTGATGAC
ERK	ACACGTTGGTACAGAGCTCCAGAA	TGCAGCCCACAGACCAAATATC
HK1	CCCTGTGGGTGTCTTGTGTG	AGGGCGCATTACTCCAGAG
LDHA	GACTGTACTTGACAATGTTGGGA	TGTCTCCAGCAAAGACTACTGT
PFKM	GCACTTCCAATCACTGTGCC	TGTGGTCCGAGTTGGTATCTT
PGAM1	CTGTCAGACCGCCATAGTGT	TCTGTGCAGAAGAGAGCAATCC
PGK1	GCTCCATTGTCCAAGCAGAAT	ATGTCGCTTTCCAACAAGCTG
PFKP	GGTCGCACGTCTCGACAAT	TGGTGCCATCATGCTATCTGA
TJP1	GCCGCTAAGAGCACAGCAA	TCCCCACTCTGAAAATGAGGA
Piezo1	TGAAGACGATAGCTGTCATCCA	TCATCATCCTTAACCACATGGTG
TRPV1	CCACTGGTGTTGAGACGCC	TCTGGGTCTTTGAACTCGCTG
